# Endogenous T cell responses to fusion-derived neoantigens in pediatric acute leukemias

**DOI:** 10.1038/s41375-025-02710-7

**Published:** 2025-07-24

**Authors:** Ricky Tirtakusuma, Mohamed A. Ghonim, Stefan Schattgen, Bradley Muller, Lee Ann Van de Velde, Tanya M. Khan, Jeremy Chase Crawford, Jing Ma, Sherif Abdelhamed, Kasi Vegesana, Walid Awad, E. Kaitlynn Allen, Ilaria Iacobucci, Charles G. Mullighan, Jeffery M. Klco, Paul G. Thomas

**Affiliations:** 1 Department of Host-Microbe Interactions, St Jude Children’s Research Hospital, Memphis, TN USA; 2 Department of Oncology, St. Jude Children’s Research Hospital, Memphis, TN USA; 3 University of Tennessee Health Science Center, Memphis, TN USA; 4 Department of Pathology, St. Jude Children’s Research Hospital, Memphis, TN USA; 5 Center of Excellence for Leukemia Studies, St. Jude Children’s Research Hospital, Memphis, TN USA

**Keywords:** Immunotherapy, Acute myeloid leukaemia

## Abstract

Pediatric patients with fusion-driven leukemias frequently have a poor prognosis and need more effective therapies. Adoptive T-cell therapies, using expanded autologous T cells, have shown promise as an immunotherapeutic for patients with tumors characterized by high mutational burdens. However, this approach has not been shown to be effective in pediatric leukemias. In this study, we analyzed samples from pediatric patients with fusion-driven acute lymphoblastic, acute myeloid, and mixed phenotypic leukemias, including those with *KMT2A*-rearrangements. T cells were attained from bone marrow samples, expanded, and their reactivity against autologous leukemic blasts was tested. Strikingly, we observed leukemia-reactive T cells in nearly all patients (33 of 34) at diagnosis or relapse. Furthermore, some patients contained clones reactive to fusion neoantigens and other tumor-associated antigens, and candidate samples were further enriched by selecting for PD1^hi^ and CD39^+^ T-cell populations. These clones were only present at the initial diagnostic timepoint and could not be detected at later times after treatment, even with deep sequence profiling. Altogether, our data suggest that adoptive T cell therapy, using expanded leukemia-reactive T cells identified at diagnosis, has potential as a novel therapeutic for these patients.

## Introduction

Fusion-driven leukemias can be clinically aggressive and associated with poor prognosis. Despite the overall improvement in outcomes for pediatric patients with acute lymphoblastic leukemia (ALL), acute myeloid leukemia (AML), and mixed phenotypic leukemia (MPAL) over the last several decades, some subtypes characterized by specific genetic fusions continue to be associated with poor outcome. For example, leukemias harboring certain *KMT2A* rearrangements can have a dismal prognosis, with a 5-year event free survival less than 30% [1]. To improve outcomes, agents that target fusion-associated pathways are currently being tested, including Menin [2] and NSD1 inhibitors [3]. Furthermore, chimeric antigen receptor T cells have been practice-changing in B-cell malignancies and have shown some promise in AML [4], but do not target fusion-specific antigens.

T-cell receptor (TCR)-engineered T cells and cancer vaccines have also shown potential. WT1 and minor histocompatibility antigens have been major candidates of these novel therapeutics [5–9] in treating AML. These approaches are currently being investigated as therapeutic strategies after disease progression or as relapse prophylaxis during remission after hematopoietic stem cell transplantation. Moreover, the combination of chemotherapy and immune-checkpoint blockade was recently introduced into AML treatment, with promising results [10]. Furthermore, the hypomethylating agent, azacitidine, enhances immune responses by increasing interferon gamma (IFN-γ) production and major histocompatibility complex (MHC) expression, and is now being used in combination with PD1 blockade in AML treatment with modest improvement in survival [11, 12]. Additional trials of combinations of nivolumab [13] and pembrolizumab [14] with conventional chemotherapy suggest that adding PD1-blockade also improves outcome. Altogether, the success of immunotherapies and other immune modulating agents in the treatment of leukemia correlates with improved adaptive immune responses against leukemic blasts, particularly by T cells.

Recently, our group observed endogenous T-cell recognition of the ETV6::RUNX1 fusion neoantigen in patients with ALL, which is the most common genetic aberration in childhood ALL, occurring in approximately 25% of patients [15, 16]. Furthermore, other studies have identified T-cell responses in leukemias that are characterized by *BCR*::*ABL1* [17] or *CBFB*::*MYH11* [18] fusions. These fusions and others that characterize different leukemic subtypes occur early in leukemogenesis, even in pre-leukemic clones, and are essential for cell survival [19–26]. For example, RNA-interference molecules and other inhibitors targeting different fusions, including *BCR::ABL1* [27, 28], *KMT2A::AFF1* [29], *KMT2A::MLLT1* [30], *RUNX1::RUNX1T1* [31], and *PML::RARA* [32], showed potent anti-leukemic activity. Together, these data suggest that targeting fusion genes has the potential to induce durable remissions.

Adoptive T-cell therapy using expanded endogenous T cells in the treatment of leukemia characterized by fusion oncoproteins has yet to be investigated. In this study, we asked whether rapid lymphocyte expansion protocols could enrich anti-tumor T cell responses in leukemias characterized by different genetic fusions. Subsequently, we investigated the reactivity of T cells to fusion-derived neoantigens, their longitudinal dynamics during treatment, and their efficacy at eliminating leukemic blasts. Additionally, we identified TCRs that responded to tumor blasts but not specific fusions. Future studies will focus on exploring a wider array of leukemia-specific mutations to better capture the full spectrum of immunogenic targets.

## Results

### T-cell expansion in leukemias characterized by genetic fusions

**Fig. 1:**
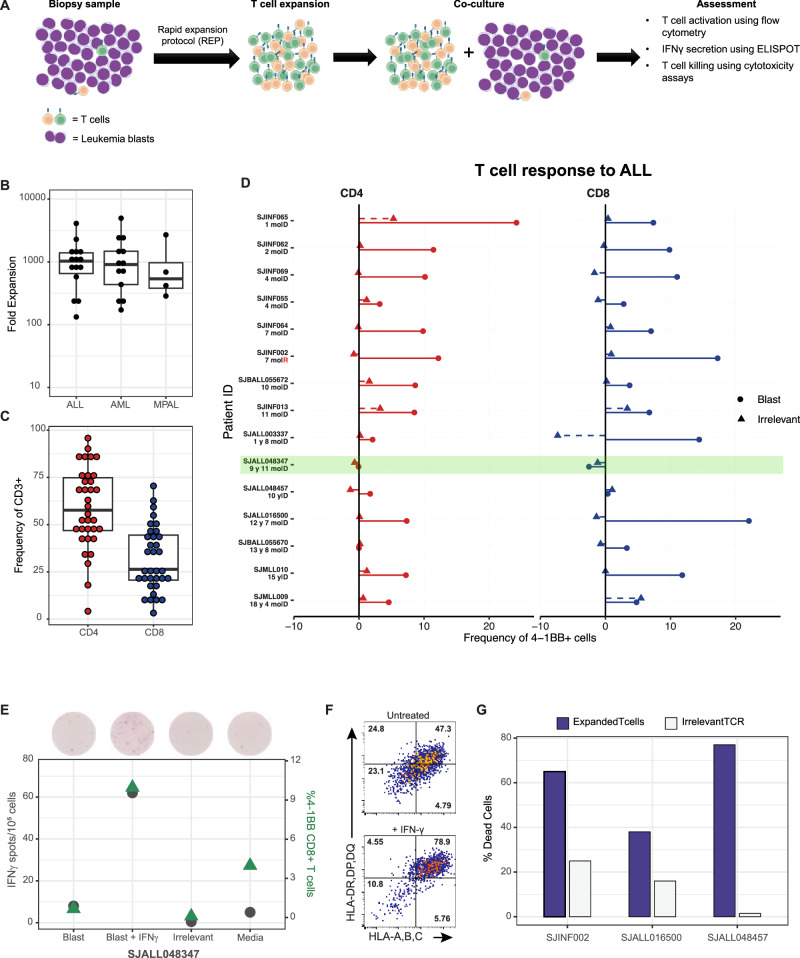
Expanded T cells and their reactivity to autologous ALL blasts. **A** Bone marrow or peripheral blood mononuclear cells biopsies, predominantly containing leukemia blasts with a minor population of autologous T cells, are initially obtained. T cells from these biopsies are expanded using the Rapid Expansion Protocol (REP), generating sufficient numbers of T cells. Subsequently, expanded T cells are co-cultured with leukemia blasts from the original biopsy. T cell reactivity against leukemia blasts is then evaluated using three complementary assays: (i) surface activation marker expression assessed by flow cytometry, (ii) IFN-γ secretion measured by ELISPOT assay, and (iii) direct cytotoxicity determined through T cell killing assay. **B** Fold expansion of T cells from ALL, AML, or MPAL bone marrow or peripheral blood samples obtained at diagnosis or relapse (see Table 1). The cell numbers were calculated after 14 days of REP in culture. **C** Percentage of CD4^+^ cells and CD8^+^ cells within the CD3^+^ T cell population among the expanded T cells of the ALL, AML, and MPAL materials. **D** Expanded T cells were co-cultured for 20 h with autologous ALL blasts, irrelevant K562 cells, or in media only as background control. The plots depict the proportion of 4-1BB cells within the CD4^+^ and CD8^+^ T cell populations, as measured by flow cytometry. The percent 4-1BB expression after T cell co-culture with ALL blasts or irrelevant cells is calculated by subtracting the background control percentage. **E** SJALL048347 blasts were pre-treated with 50 ng/mL IFN-γ for 72 h. Samples (untreated and pre-treated), irrelevant control, and media-only were co-cultured with the expanded T cells for 20 h. T-cell response was measured by IFN-γ ELISPOT (left axis) and flow cytometry on the CD8^+^ 4-1BB^+^ population (right axis). **F** Flow analysis of MHC class I and II expression on SJALL048347 upon IFN-γ treatment. **G** Lysis of autologous blasts by expanded T cells from samples SJINF002, PDX SJALL016500, and SJALL048457 after an 18 h incubation, as measured by flow cytometry. An irrelevant TCR control was used, consisting of T cells expressing a TCR recognizing RAS G12V mutation (KLVVVGAVGV).

Blood (*n* = 7) or bone marrow (*n* = 27) samples were obtained from 34 patients at the time of diagnosis or relapse. The cohort included 15 patients with ALL, 15 patients with AML, and 4 patients with MPAL. Eight patients with ALL were infants with the *KMT2A*::*AFF1* fusion. Samples were curated based on whether their genetic mutations were predictors of poor prognosis (e.g., *KMT2A*-rearrangement, *NUP98*::*NSD1*, *PICALM*::*MLLT10*, or *DEK*::*NUP214*), were highly prevalent (*RUNX1*::*RUNX1T1*), or were enriched in MPAL (e.g., ZNF384::*EP300* in B/myeloid MPAL [33]) (Table 1). The compositions of bone marrow and peripheral blood were dominated by leukemic blasts (median, 92%; range, 59–99%), and the median frequency of lymphocytes was only 3.5% (range, 1–25%) (Table 1). To adequately assess T-cell reactivity, we expanded the number of T cells using a rapid expansion protocol (REP) and cultured them for 14 days to overcome the low lymphocyte cellularity (Fig. 1A). Amplification was successful in all samples, irrespective of genetic function or lymphocyte count. T-cell expansion ranged from 133- to 4092-fold increase (median, 1027-fold) in ALL samples, 172- to 4957-fold increase (median, 907-fold) in AML, and 286- to 2693-fold increase (median, 554-fold) in MPAL (Fig. 1B). The median frequencies of CD4^+^ and CD8^+^ T cells after the expansion, compared with that of CD3^+^ T cells, were 59.7% and 26.1%, respectively (Fig. 1C).

**Table 1:** Clinical characteristics of pediatric patients with acute leukemias harboring genomic fusions.

	Patient	Sex	Age at diagnosis	Disease stage	Sample source	Disease	Fusion gene	WBC (x1000/mm^3^)	% Blast	% Lymphocyte	CNS
1	SJINF002	M	7 mo	Relapse	Bone marrow	ALL	KMT2A::AFF1	116.6	92	1	Unk
2	SJINF013	F	11 mo	Diagnosis	Bone marrow		KMT2A::AFF1	62.6	96	2	Unk
3	SJINF064	F	7 mo	Diagnosis	Bone marrow		KMT2A::AFF1	60.4	95	<1	Unk
4	SJINF065	M	1 mo	Diagnosis	Bone marrow		KMT2A::AFF1	906	99	<1	Unk
5	SJINF055	F	4 mo	Diagnosis	Bone marrow		KMT2A::AFF1	1136	97	<1	Unk
6	SJINF062	F	2 mo	Diagnosis	Bone marrow		KMT2A::AFF1	106	96	2	Unk
7	SJINF069	F	4 mo	Diagnosis	Bone marrow		KMT2A::AFF1	83	93	1	Unk
8	SJBALL055672	M	10 mo	Diagnosis	Peripheral blood		KMT2A::AFF1	109.6	92	4	Unk
9	SJMLL009	M	18 y 4 mo	Diagnosis	Bone marrow		KMT2A::AFF1	17.1	59	1	Unk
10	SJMLL010	M	15 y 0 mo	Diagnosis	Bone marrow		KMT2A::AFF1	138.6	99	<1	Unk
11	SJALL048347	M	9 y 11 mo	Diagnosis	Peripheral blood		KMT2A::AFF1	897	96	2	Y
12	SJBALL055670	M	13 y 8 mo	Diagnosis	Bone marrow		KMT2A::AFF1	NA	90	7	Unk
13	SJALL003337	F	1 y 8 mo	Diagnosis	Bone marrow		KMT2A::AFF1	NA	N/A	N/A	Unk
14	SJALL016500	M	12 y 7 mo	Diagnosis	Bone marrow		KMT2A::MLLT10	14.3	88	5	Unk
15	SJALL048457	F	10 y 0 mo	Diagnosis	Bone marrow		PICALM::MLLT10	180.9	87	2	N
16	SJAML030440	M	3 y 0 mo	Diagnosis	Bone marrow	AML	KMT2A::MLLT3	NA	N/A	N/A	Unk
17	SJAML001441	M	11 y 9 mo	Diagnosis	Bone marrow		NUP98::NSD1	101	93	3	Unk
18	SJAML030547	M	3 y 3 mo	Diagnosis	Bone marrow		RUNX1::EVX1	142.1	83	1	Y
19	SJAML030669	M	18 y 4 mo	Diagnosis	Bone marrow		RUNX1::RUNX1T1	5.7	60	17	N
20	SJAML030471	F	16 y 11 mo	Diagnosis	Bone marrow		RUNX1::RUNX1T1	17.1	93	1	N
21	SJAML030459	M	11 y 9 mo	Diagnosis	Bone marrow		PICALM::MLLT10	NA	N/A	13	Unk
22	SJAML043616	F	7 y 7 mo	Relapse	Peripheral blood		KMT2A::MLLT1	6.2	93	5	Unk
23	SJAML060226	F	13 y 2 mo	Diagnosis	Bone marrow		KMT2A::MLLT1	NA	91	4	N
24	SJAML004919	F	12 y 9 mo	Diagnosis	Bone marrow		KMT2A::MLLT3	91.9	70	3	Unk
25	SJMLL014	F	14 y 7 mo	Diagnosis	Bone marrow		KMT2A::MLLT3	91.8	93	4	Unk
26	SJAML061496	M	14 y 9 mo	Diagnosis	Bone marrow		KMT2A::MLLT4	128.1	74	6	Unk
27	SJAML031669	F	9 y 0 mo	Diagnosis	Bone marrow		KMT2A::MLLT4	NA	N/A	N/A	Unk
28	SJAML056339	M	13 y 7 mo	Diagnosis	Peripheral blood		KMT2A::MLLT6	87.7	90	9	Unk
29	SJAML005142	M	17 y 9 mo	Relapse	Bone marrow		KMT2A::MLLT10	207.9	N/A	25	Unk
30	SJMLL012	M	11 y 8 mo	Diagnosis	Peripheral blood		KMT2A::MLLT10	319.6	98	2	Unk
31	SJMPAL016107	F	6 y 1 mo	Diagnosis	Bone marrow	MPAL	MLLT10::PICALM	63.4	59	14	Unk
32	SJMPAL011911	M	15 y 8 mo	Diagnosis	Peripheral blood		BCL11B enhancer tandem amplification	141.9	89	2	Unk
33	SJMPAL012424	M	8 y 4 mo	Diagnosis	Bone marrow		KMT2A::MLLT3	288.4	88	7	Unk
34	SJMPAL012425	F	14 y 0 mo	Diagnosis	Peripheral blood		ZNF384::EP300	NA	N/A	N/A	Unk

### T-cell recognition and reactivity against autologous ALL blasts

We examined whether the expanded T cells recognize and react to autologous leukemic blasts by measuring IFN-γ secretion and upregulation of co-stimulatory receptors 4-1BB and OX40 after overnight co-culture, as previously reported [34, 35]. An increase in 4-1BB expression on both CD8^+^ and/or CD4^+^ T cells was observed in 13 of 15 ALL samples (Fig. 1D, Supplementary Fig. 1). Moreover, OX40 was highly upregulated on CD4^+^ cells and IFN-γ secretion was increased, as determined by ELISPOT assay (Supplementary Fig. 2A). Although we did not observe a robust upregulation of the activation marker on CD8 in one sample (SJMLL009), IFN-γ secretion was markedly increased compared to that in the irrelevant control (119 spots in blast co-culture, 7 spots in irrelevant control, and 0 spots in T cells only control; Supplementary Fig. 2B).

Of the 15 ALL samples, only SJALL048347 did not show a T-cell response (Fig. 1D, Supplementary Fig. 2A). We hypothesize that the blasts in that sample might have lower MHC expression or antigen presentation that could be induced by IFN-γ exposure, as previously reported [36, 37]. To that end, we pre-treated leukemic blasts with 50 ng/mL of IFN-γ for 72 hours, washed them, and then co-cultured them with autologous T cells. T-cell reactivity was remarkably increased, as indicated by the upregulation of 4-1BB and IFN-γ secretion (Fig. 1E). This response was associated with a simultaneous 2.6-fold and 1.8-fold upregulation in MHC class I and II, respectively (Fig. 1F, Supplementary Fig. 3).

To further expand on these findings, we assessed T-cell killing by the expanded T cells of the autologous blasts in an infant (less than 1 years of age) ALL sample with *KMT2A*::*AFF1* (SJINF002), a pediatric ALL sample with *KMT2A*::*AFF1* (SJALL016500; patient derived xenograft - PDX), and a pediatric ALL sample with *PICALM::MLLT10* (SJALL048457). All 3 samples demonstrated effective T cell-mediated killing of autologous leukemic blasts (Fig. 1G), suggesting T-cell reactivity in all 15 patients with ALL.

### T-cell recognition and reactivity against autologous AML and MPAL blasts

**Fig. 2:**
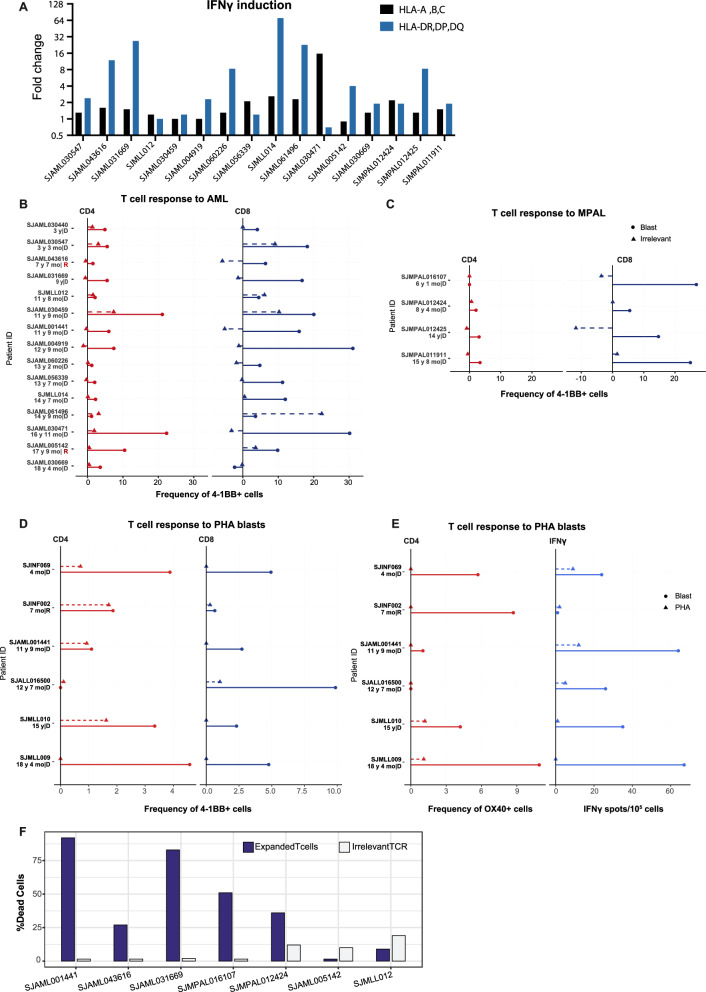
Expanded T-cell reactivity to autologous AML or MPAL blasts. **A** Fold change of MHC class I and II expression on AML and MPAL blasts upon IFN-γ treatment. Leukemia blasts were treated with 50 ng/mL IFN-γ for 72 h. Untreated material was prepared as the control. **B**, **C** Expanded T cells were co-cultured for 20 h with autologous AML (**B**) or MPAL (**C**) blasts, irrelevant (K562) cells, or media/T cells only as background control. The plots depict the proportion of 4-1BB cells within the CD4^+^ and CD8^+^ T cell populations, as determined by flow cytometry. The values for T cell co-cultures with ALL blasts and irrelevant cells are presented after subtracting the background control values. **D**, **E** Expanded T cells were co-cultured for 20 h with autologous blasts, PHA blasts, or media only as background control. The plots depict the proportion of 4-1BB expressing cells within the CD4^+^ and CD8^+^ cell populations (**D**), OX40^+^ expression of CD4^+^ cells, and IFN-γ secretion (**E**). The values are presented after subtracting the background control values. **F** Lysis of autologous blasts by expanded T cells from AML samples SJAML001441 (assessment on PDX blasts), SJAML043616, SJAML031669, SJAML005142, and SJMLL012 and from MPAL samples SJMPAL016107 and SJMPAL012424 after 18 h incubations, as measured by flow cytometry. An irrelevant TCR control was used, consisting of T cells expressing a TCR recognizing RAS G12V mutation (KLVVVGAVGV).

We next evaluated T-cell responses in 15 AML and 4 MPAL samples. These samples included *KMT2A*-rearrangements, *NUP98::NSD1*, *PICALM::MLLT10*, *RUNX1:RUNX1T1*, and *ZNF384::MLLT10* molecular alterations (Table 1). The MPAL cases consisted of 2 patient samples with T/myeloid phenotypes (SJMPAL016107 and SJMPAL011911) and 2 patients with B/myeloid phenotypes (SJMPAL012424 and SJMPAL012425). We used the REP culture system to expand the T cells and then co-cultured the expanded T cells with autologous leukemia blasts. Unlike the ALL blasts, the AML and MPAL blasts required IFN-γ treatment prior to co-culturing. Without the pre-exposure to IFN-γ, most samples showed either minimal or no T-cell reactivity (Supplementary Fig. 4). We then evaluated MHC class I and II expression after IFN-γ pre-treatment. Both MHC class I and II were upregulated at a median of 1.4-fold (range, 0.9 to 15.7-fold) and 2.4-fold (range, 0.7 to 70.5-fold), respectively (Fig. 2A, Supplementary Figs. 5, 6).

The expanded T cells were co-cultured with their respective autologous IFN-γ treated AML or MPAL blasts; 4-1BB was upregulated on CD8^+^ and/or CD4^+^ populations in 13 AML cases, which correlated with an increase in IFN-γ production and OX40 expression by CD4^+^ T cells (Fig. 2B, Supplementary Fig. 7A). Like the ALL sample SJMLL009, the AML sample SJMLL012 showed T cell reactivity by IFN-γ secretion and OX40 on CD4^+^ T cells, despite no upregulation of the 4-1BB marker (Fig. 2B, Supplementary Fig. 7B). Of the 15 AML samples, only SJAML061496 showed no reactivity. While all MPAL samples showed activation marker upregulation, the most consistent and robust signal was observed for 4-1BB in CD8^+^ T cells, with variable levels of 4-1BB and OX40 expression in CD4^+^ T cells (Fig. 2C, Supplementary Fig. 7C).

To further substantiate the specificity of T cell responses, we included autologous PHA blasts generated from remission bone marrow as an additional negative control. PHA blasts were produced for samples SJINF069, SJINF002, SJAML001441, SJALL016500, SJMLL010, and SJMLL009. We demonstrated that T cell responses, as indicated by activation markers and IFN-γ secretion, were substantially higher when co-cultured with autologous leukemia blasts compared to PHA blasts (Fig. 2D, E). The lack of reactivity against PHA blasts confirms the specificity of the observed T cell responses, ensuring that they were directed against leukemic cells rather than normal activated lymphocytes.

Next, we assessed the T-cell killing of the autologous blasts in 5 AML samples and 2 MPAL samples. Three of the 5 AML samples and both MPAL samples showed cytotoxicity (Fig. 2F). Both AML samples with the *KMT2A*::*MLLT10* fusion gene showed no cytotoxicity (SJAML005142 and SJMLL012, Fig. 2F).

### Expression of PD1 or CD39 marks leukemia blasts for T-cell recognition

Recent work in solid tumors, including breast [38], head and neck [39], endometrial [40], and non–small cell lung cancer [41, 42], showed that PD1 and CD39 mark tumor-reactive T cells. Furthermore, PD1 and CD39 can be used to specifically select and expand tumor-reactive T cells [40]. Here we investigated whether these markers also identify leukemia-reactive T cells.

**Fig. 3:**
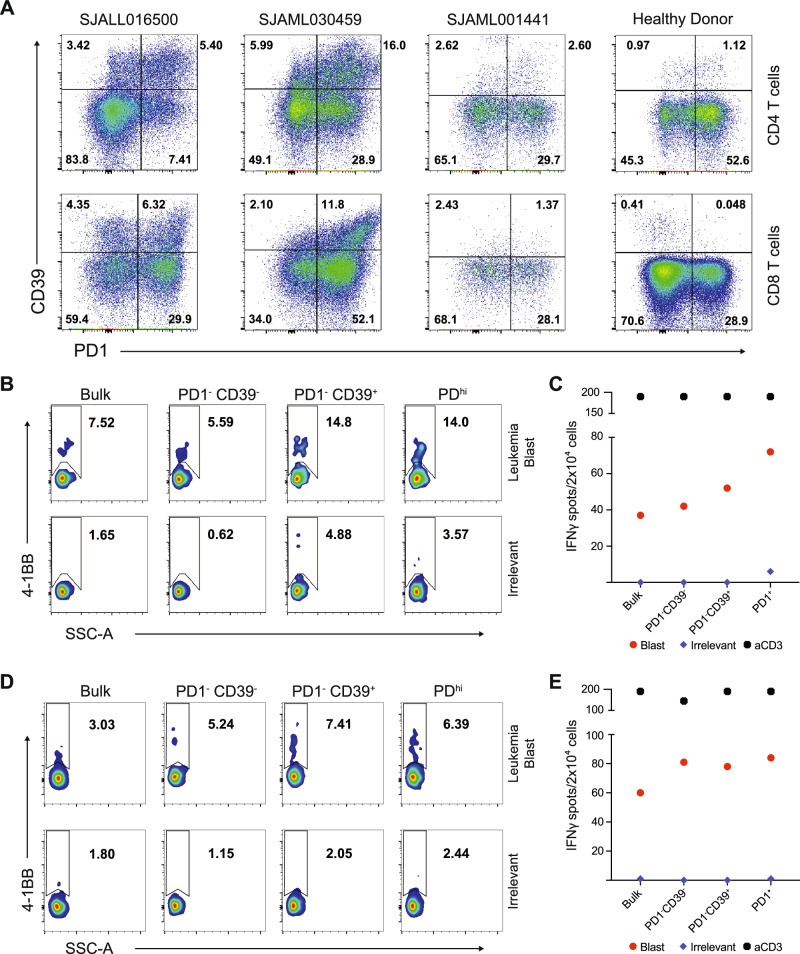
Reactivity of CD39^+^ and PD1^+^ T-cell subsets to leukemia blasts. **A** Flow cytometry plots of leukemia blasts from samples SJALL016500, SJAML030459, SJAML001441, and a healthy donor control. The cells were gated for CD19^–^ or CD33^–^ for ALL or AML, respectively, and for CD3^+^ CD4^+^ (top panel) and CD8^+^ (bottom panel). **B**, **C** T-cell subsets were sorted and expanded for 14 days and then co-cultured with autologous blasts from sample SJALL016500 for 20 hours. T-cell recognition was measured by CD8^+^ 4-1BB^+^ expression (**B**) and IFN-γ ELISPOT (**C**). **D**, **E** T-cell subsets were sorted, expanded, and then co-cultured with autologous blasts from sample SJAML030459, as in (**B**, **C**). T-cell recognition was measured by CD8^+^ 4-1BB^+^ expression (**D**) and IFN-γ ELISPOT (**E**).

We evaluated 3 diagnostic samples, including ALL with *KMT2A::MLLT10* (SJALL016500), AML with *PICALM::MLLT10* (SJAML030459), and AML with *NUP98::NSD1* (SJAML001441). All samples included a population of T cells that was PD1^+^ CD39^+^, but it was smaller in SJAML001441 (Fig. 3A). T cells were isolated based on the markers they expressed (*i.e*., PD1^–^ CD39^–^, PD1^–^ CD39^+^, and PD1^hi^) and then expanded in vitro. The expanded T cells were then co-cultured with autologous blasts to assess their reactivity. PD1^–^ CD39^+^ and PD^hi^ populations had up to 2.5-fold greater response to leukemia blasts compared to the irrelevant control, as shown by 4-1BB upregulation and increased IFN-γ secretion (Fig. 3B–E, Supplementary Fig. 8). Furthermore, consistent with a previous report, the PD1^hi^ population co-expressed CD39 and TIM3 [40], at higher levels than did the PD1^dim^ or PD1^–^ populations (Supplementary Fig. 9). CD39 and TIM3 were more highly expressed on T cells from our leukemia cohort, when compared to cells from healthy donors (Supplementary Fig. 10).

### Identification of leukemia blast- and fusion gene-reactive T-cell receptors

**Fig. 4:**
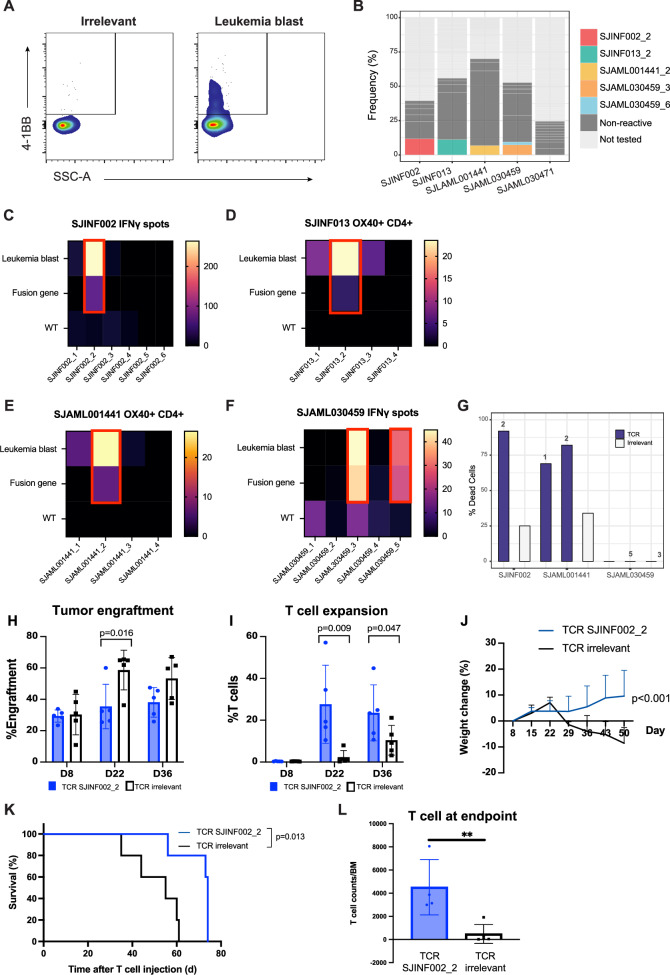
TCR reactivity to leukemia blasts and fusion gene neoantigen. **A** Representative sorting method for single-cell TCR sequencing on CD8^+^ T cells. Expanded T cells were co-cultured with the autologous blasts or irrelevant (K562) cell control. T cells were gated for CD3^+^, CD8^+^, or CD4^+^ and the irrelevant control was used to gate for the negative population-activation marker. **B** Frequency TCR clonotypes from (**A**) single-cell sorting (see Supplementary Table 1). The top-ranked clonotypes by frequency were assessed for their reactivity to leukemia blasts and fusion genes presented by APCs. Colored bars indicate clonotypes that were reactive to the blasts or fusion gene; dark gray bars indicate nonreactive clonotypes; and light gray bars indicate clonotypes that were not tested. **C–F** Top-ranked TCRs by frequency were over-expressed on TRAC knockout allogeneic healthy donor cells. They were co-cultured with primary leukemia materials to evaluate the reactivity to the blasts. Secondly, antigen-presenting cells from the patients were electroporated with their corresponding fusion gene in RNA minigene format. B cells were used as APCs for sample SJINF013, and CD4^+^ T cells for other samples. TCR’s specificity to the fusion genes was assessed by coculturing with the electroporated cells. The samples include (**C**) SJINF002, (**D**) SJINF013, (**E**) SJAML001441, and (**F**) SJAML030459. The fusion genes were *KMT2A* exon 10–*AFF1* exon 4 (SJINF002), *KMT2A* exon 10–*AFF1* exon 5 (SJINF013), *NUP98* exon 11–*NSD1* exon 6 (SJAML001441), and *PICALM* exon 19–*MLLT10* exon 4 (SJAML030459). Wild type (WT) refers to the germline *KMT2A*, *NUP98*, and *PICALM* sequences, respectively. **G** Reactive TCRs SJINF002_2, SJAML001441_1, SJAML001441_2, SJAML030459_3, and SJAML030469_6 were co-cultured with leukemia blasts for 18 h, and blast lysis was measured by flow cytometry. TCR clonotype was listed above the TCR bar. Leukemia burden and T cells were monitored by FACS on blood samples. The frequencies of engraftment (**H**) and reactive-T cell expansion (**I**) on weeks 1, 3, and 5 were plotted. Each dot represents an independent mouse. *P* value based on a Kruskal-Wallis test. **J** Mouse body weight changes were shown relative to day 0 after T-cell transfer. *P* value based on linear regression analysis. **K** Kaplan-Meier survival curve of NSG mice treated with TCR SJINF002_2 or irrelevant control. Survival differences were analyzed using the log-rank test. **L** T cells expressing TCR SJINF002_2 persisted in the bone marrow at the time of euthanasia.

To identify the specific T-cell receptors (TCRs) that recognize leukemic blasts and fusion gene-derived neoantigens, the expanded T cells were co-cultured with autologous blasts, and then the leukemia-reactive T cells were enriched by sorting for 4-1BB^+^ on the CD8^+^ population [43] and for 4-1BB^+^, OX40^+^, or 4-1BB^+^ OX40^+^ on the CD4^+^ population [44] (Fig. 4A). Cells were sorted into a 384-well plate for single-cell TCR sequencing. We included infant ALL samples with a *KMT2A::AFF1* fusion at diagnosis (SJINF013), or at relapse (SJINF002) and AML samples with *PICALM::MLLT10* (SJAML030459), *NUP98::NSD1* (SJAML001441), or *RUNX1::RUNX1T1* (SJAML030471) fusions. Importantly, infant ALL with *KMT2A*-rearrangements has a mean of 1.3 non-silent mutations [45], which lowers the complexity of the neoantigen screening.

Single-cell TCR sequencing of the sorted populations showed the presence of major clonotypes, defined as greater than 8% frequency within the sorted population, in 4 (SJINF002, SJINF013, SJAML030459, SJAML001441) of the 5 samples (Supplementary Table 1). We selected the top 4–6 clonotypes in these samples to evaluate their reactivity to leukemia blasts and their fusion genes. Additionally, we selected the top 10 clonotypes of SJAML030471, which did not show any major reactive expansions. We utilized healthy donor peripheral blood lymphocytes as an alternative to autologous peripheral blood mononuclear cells (PBMCs). Endogenous TCRs on healthy donor peripheral blood lymphocytes were knocked out by using CRISPR of the *TRAC* locus [46] prior to overexpression of the candidate TCRs via retroviral transduction (Supplementary Fig. 11). Fusion breakpoint sequences were obtained by PCR analysis of cDNA, followed by Sanger sequencing (Supplementary Fig. 12). Fusion genes were overexpressed on their corresponding antigen-presenting cells (APCs) and co-cultured with peripheral blood lymphocytes containing our candidate TCRs. B cells were generated from a PBMC remission sample (sample SJINF013) to serve as APCs. CD4^+^ T cells were used in other samples due to the unavailability of remission samples. We identified TCR SJINF002 clone 2 (ranked 2 by frequency; SJINF002_2), SJINF013 clone 2 (SJINF013_2), SJAML001441 clone 2 (SJAML001441_2), and SJAML030459 clones 3 and 5 (SJAML030459_3 and SJAML030459_5), as reactive to leukemic blasts and/or fusion neoantigens (Fig. 4C–F, Supplementary Figs. 13–15). The reactive clonotypes were not necessarily the most abundant clonotypes in the sorted population (Supplementary Table 1), which has been previously reported [43]. Despite not showing reactivity to the fusion neoantigen, the most prevalent TCR clonotype of SJINF013 and SJAML001441 (TCR SJINF013_1 and SJAML001441_1, respectively) showed reactivity to leukemic blasts (Fig. 4D, E). Furthermore, sample SJAML030471, which did not have any major clonotypes, lacked TCR reactivity to both fusion neoantigen and leukemic blasts (Supplementary Fig. 13).

### Leukemia blast- and fusion gene-reactive TCRs exhibit antileukemic activity in vitro and in vivo

We confirmed the antigen recognition of the reactive TCRs by performing cytotoxicity assays. First, we tested the reactivity against primary leukemic blasts from SJINF002 and SJAML001441, and PDX SJAML030459 (due to limited sample availability) in vitro. Peripheral blood lymphocytes transduced with reactive TCRs SJINF002_2, SJAML001441_1, SJAML001441_2, SJAML030459_3, and SJAML030459_5 were co-cultured with their respective leukemia blasts, and elimination of blasts was assessed. We observed T cell-mediated killing of leukemic blasts in SJIN002_2, SJAML001441_1, and SJAML001441_2 but not in SJAML030459_3 and SJAML030459_5 (Fig. 4G). A likely explanation is that the PDX cells did not proliferate robustly in vitro, which may have led to reduced expression of antigen presentation machinery and fusion antigens themselves, thereby limiting T cell recognition and cytotoxicity. Notably, the most prevalent TCR clonotype SJAML001441 (TCR SJAML001441_1) was cytotoxic against leukemic blasts, despite not being reactive towards the fusion neoantigen, indicating recognition of another tumor-specific or associated antigen.

We next evaluated the reactivity of TCR SJINF002_2 in vivo. NSG mice were transplanted with 10^6^ blasts from SJINF002 and monitored for engraftment by weekly blood draws and performing fluorescence-activated cell sorting (FACS). T cells expressing a reactive TCR (SJINF002_2) or irrelevant control were generated by retroviral overexpression in TRAC KO PBMCs from healthy controls (Supplementary Fig. 16) once engraftment was observed (0.1% blasts). Then, 5 × 10^6 ^T cells expressing SJINF002_2 were intravenously infused. Tumor burden and persistence of the transferred T cells in peripheral blood were monitored weekly, along with body weight as it’s an indicator of morbidity. We observed T cell expansion and significantly reduced tumor burden in SJINF002_2 TCR-treated mice starting at week 3 post T cell transfer (Fig. 4H, I), which also correlated with significantly higher mean body weight (Fig. 4J) and improved survival (Fig. 4K) compared to control animals. Moreover, the SJINF002_2 TCR T cells were detectable in the bone marrow till their defined end points (range 9–12 weeks) (Fig. 4L).

### Characterization of fusion gene-reactive TCRs

**Fig. 5:**
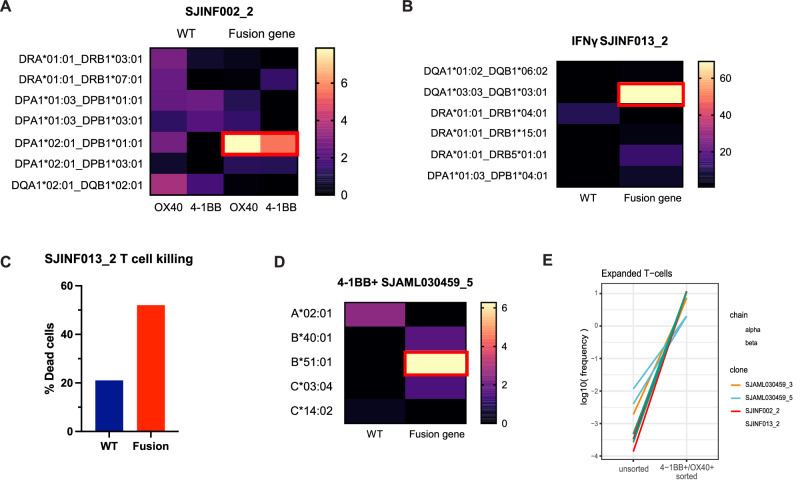
HLA-restriction of Reactive TCRs. **A–D** Reactive TCRs were co-cultured with 293 T CIITA or K562 cells expressing individual HLA alleles from the corresponding patients, serving as APCs. The RNA-fusion gene or wild-type sequence was overexpressed on the APCs. T cell reactivity was measured by flow cytometry, assessing OX40 and 4-1BB upregulation, or by IFN-γ ELISPOT. **A** TCR SJINF002_2 showed restriction to HLA-DPA1*02:01/DPB1*01:01 complex. **B** TCR SJINF013_2 exhibited reactivity to HLA-DQA1*03:03/DQB1*03:01 as indicated by IFN-γ spots (**B**) and confirmed by T cell-mediated killing of K562 cells expressing the HLA-DQA1*03:03/DQB1*03:01 fusion sequences (**C**)**. D** TCR SJAML030459_5 demonstrated reactivity to HLA-B*51:01. **E** Frequencies of the reactive TCRs were compared between the unsorted expanded T cells and those sorted following co-culture with autologous blasts. A higher frequency of reactive TCRs was observed only in the sorted T cells expressing activation markers.

We examined the human leukocyte antigen (HLA)-restricted elements that presented the fusion neoantigens. HLA typing was performed (Supplementary Table 2), and individual MHC II alleles were transiently transfected into the 293T-CIITA cell line with fusion gene RNA. Our sequencing identified the *KMT2A::AFF1* fusion from patient SJINF002 with the breakpoint located at exons 10 and 4, respectively (Supplementary Fig. 12). This fusion was recognized by TCR SJINF002_2 when presented by DPA1*02:01/DPB1*01:01 (Fig. 5A). This result was confirmed using the K562 artificial antigen-presenting cell model as an alternative antigen-presentation source [15]. The K562 cell line was transduced with MHC class II machinery, including CD64, CD80, CD83, CD74, and HLA-DM, using a lentivirus vector. Individual MHC allele, fusion gene, or wild-type control sequences were transfected into the cell line and maintained in co-culture with the candidate TCR. We observed consistent results of TCR reactivity to the *KMT2A*::*AFF1* fusion neoantigen when presented by DPA1*02:01/DPB1*01:01 (Fig. 5A).

Similarly, for sample SJINF013, the fusion breakpoint is located at *KMT2A* exon 10 and *AFF1* exon 5 (Supplementary Fig. 12). We screened the reactive TCR SJINF013_2 against its corresponding HLAs and confirmed its restriction to HLA-DQA1*03:03/DQB1*03:01 complex (Fig. 5B). This specificity was further validated by a cytotoxicity assay. The fusion gene or wild-type control was overexpressed in the engineered K562 cell line containing this HLA allele and then co-cultured with TCR SJINF013_2. The TCR demonstrated cytotoxic reactivity specifically against the fusion gene presented by this allele (Fig. 5C).

HLA-restriction was also determined for reactive TCR clone SJAML030459_5. Index FACS indicated this clone originated from CD8 + T cells. Each class I HLA allele for SJAML030459 and the *PICALM*::*MLLT10* fusion were transfected into 293T-CIITA and co-cultured with SJAML030459_5-expressing T cells. We observed the reactivity of TCR clone SJAML030459_5 when the fusion neoantigen, but not WT gene, was presented on HLA B*51:01 as indicated by 4-1BB upregulation (Fig. 5D).

Subsequently, we tracked the abundance of these clonotypes across time points. For TCR SJINF002 clone 2 we examined diagnosis (Day 0), first remission (Day 198), relapse (Day 1006), and remission after relapse (Day 2238). The TCR SJINF002 clone 2 was present only at relapse (Day 1006), but at a low frequency (0.001%) (Supplementary Fig. 17). Notably, this clonotype was enriched to 11.7% in the 4-1BB^+^ OX40^+^ population (Fig. 5E). We then evaluated leukemia-reactive clonotypes longitudinally in samples SJINF013 and SJAML030459. Similarly, they were only found at low frequencies and only at the diagnostic timepoint, including 0.0062% (SJINF013 clone 2), 0.001% (SJAML030459 clone 3), and 0.001% (SJAML030459 clone 5) (Fig. 5E, Supplementary Fig. 17). None of the clonotypes were detected at any later timepoints.

## Discussion

Despite the low mutational burden and neoantigen expression in pediatric leukemia, we found leukemia-reactive T cells in nearly all samples (33 of 34, 97%), regardless of leukemia subtype, genomic fusion, or demographic factors using a rapid expansion T cell protocol. Our data shows that T cells can engage and eliminate leukemic blasts, both by recognition of fusion neoantigens and other unknown antigens. Leukemia is frequently found in tissues where T cells reside, and this proximity is conducive for frequent engagement between T cells and leukemic blasts. Notably, the tumor-reactive clones identified were only found at diagnostic or relapse time points, despite deep sequencing of samples from other time points during treatment. This finding suggests current treatments may limit the expansion of these potentially protective responses.

We chose to use bone marrow aspirates as the T cell source for two reasons. Firstly, bone marrow from patients with breast cancer has been shown to contain memory T cells that are reactive to tumor-associated antigens [47]. We hypothesized that this would also apply to bone marrow from patients with leukemia. Secondly, T cells in bone marrow potentially infiltrate that niche because they recognize the antigens on the leukemic blasts therein. Although peripheral blood and bone marrow contain naïve T cells, we reasoned that bone marrow T cells are further enriched for selected antigen-experienced T cells, and our results show that neoantigen-specific T cells can be readily attained from bone marrow.

Not only did we observe T cells reactive to autologous leukemic blasts, but we identified specific TCRs that recognized fusion gene-derived neoantigens, particularly, *KMT2A::AFF1* and *PICALM::MLLT10*. These results were validated using multiple approaches to present defined neoantigen-derived epitopes on patient-matched HLAs including using patient APCs, K562 cells, and 293T-CIITA cells. *KMT2A::AFF1* occurred in 44–49% of infant and pediatric KMT2A-rearranged ALL cases. The *KMT2A* breakpoint cluster primarily localizes between *KMT2A* exon 9 and intron 11, covering 93.5% of breakpoints in acute leukemia [48]. Both SJINF002 (*KMT2A* exon 10, *AFF1* exon 4) and SJINF013 (*KMT2A* exon 10, *AFF1* exon 5) breakpoints are located within this cluster. Meanwhile, *NUP98::NSD1*, *PICALM::MLLT10*, and *RUNX1::RUNX1T1* have a conserved breakpoint, representing 7.45%, 0.77%, and 13.64% of pediatric AML, respectively [49]. The HLA-DPA1*02:01-DPB1*01:01 haplotypes present KMT2A::AFF1 with a frequency of 4.7% [50], while HLA-B*51:01 presents PICALM::MLLT10 in approximately 6% of the population [51].

Our results confirm that T cells attained from the bone marrow can express TCRs specific against fusion gene-derived neoantigens. Additionally, we showed that a TCR targeting *KMT2A::AFF1* could contribute to leukemia control in vivo against the autologous tumor. Altogether, these data show that the adaptive immune system is capable of targeting these unique tumor proteins, and it is reasonable that vaccines and other therapies manufactured to target fusion neoantigens may be efficacious.

Although cancer vaccines have been used to treat solid tumors [52–55], there has been a paucity of these tested in hematological malignancies. Fusion genes have broadly been described as essential for leukemia cell survival and propagation. Furthermore, these blasts characteristically downregulate their fusion-derived oncoproteins, thereby providing a means to immune escape. Thus, vaccines targeting these fusion-derived neoantigens may prevent this phenomenon by expanding T cell populations that target these unique antigens. Although further investigation is necessary, administering vaccines that target these fusion gene-derived oncoproteins during disease progression or as relapse prophylaxis may improve outcomes.

Given that most T cells are MHC-class restricted, we hypothesized that increasing MHC expression on blasts would improve T cell reactivity and elimination. Christopher et al. (2018) [36] previously showed that IFN-γ treatment of AML blasts at relapse increased MHC class II expression. Consistent with this finding, T cell responses to AML and MPAL were dependent on IFN-γ exposure before co-culture. Interestingly, elimination of ALL blasts by autologous T cells was successful without IFN-γ treatment. This difference may reflect the differential programing of a lymphoblast, which could have the characteristics of an APC, especially if its transcriptome is like that of a B cell. Surprisingly, we observed less T cell reactivity to autologous blasts in some ALL samples after IFN-γ treatment (SJINF013, SJINF069, SJMLL010, SJBALL055670), when compared to untreated blasts (Supplementary Fig. 18). We must emphasize, however, that this occurred only in ALL and not in AML or MPAL. Future investigations are needed to better elucidate the mechanism behind this phenomenon.

Identifying tumor-reactive T-cell signatures enables the enrichment and rapid development of TCR immunotherapies. Recent reports suggest that PD1, CD39, and CXCL13 mark tumor-reactive T cells [38–42, 56]. Our findings were consistent with those of a previous report that examined the reactivity of PD1^hi^ and CD39^+^ T cell populations and the co-expression of TIM3 in the PD1^hi^ T-cell subset [40].

TIM3 is an immune-checkpoint receptor [57, 58], and its inhibition can improve anti-tumor immune responses [59, 60]. TIM3 is recognized as a marker of leukemia-initiating cells in AML but is not expressed on hematopoietic stem cells [61, 62]. Its ligand, Galectin9, is also expressed by AML blasts, thereby creating an autocrine loop that ultimately stimulates self-renewal and propagation of the leukemia-initiating cells [62]. Additionally, TIM3 is expressed on ALL blasts, with increased expression at relapse [63]. Inhibition of this receptor may prove to be efficacious, with sabatolimab and cobolimab currently being studied in clinical trials [59, 60]. In future studies, we plan to evaluate whether TIM3 blockade can restore T cell-mediated cytotoxicity in non-responsive samples such as SJAML005142 and SJMLL012 (Fig. 2F). In combination with other immunotherapies, TIM3 blockade may further improve adaptive immune responses against leukemic blasts.

While we focused on fine mapping of fusion-derived neoantigens in this study, we were limited in that we did not correlate immune responses to the broad mutational profiles of these pediatric leukemias. Although pediatric malignancies tend to have fewer mutations than adult malignancies, the mutations can cause unique neoantigens to be created and therefore targeted by adaptive immune responses. These mutations could also impact malignant characteristics including cell survival and proliferation. Furthermore, it might be advantageous to take a pooled approach when identifying TCRs reactive against different neoantigens or when creating a cancer vaccine. These approaches would enable the targeting of a variety of antigens simultaneously, as the historical dogma of leukemia treatment has been using multi-agent chemotherapy to interfere with multiple cellular pathways simultaneously.

Moreover, it is critical to note the risk of expanding self-reactive TCRs. Because the number of mutations in pediatric leukemia is low, our TCR isolation methodology has a risk of capturing self-reactive TCRs. It is crucial to validate that the isolated TCRs are tumor-specific and non-self-reactive. We emphasize that due to the availability of the materials, we have not conducted T cell cytotoxicity to patient autologous healthy cells, e.g., CD34^+^. This limitation must be considered when proceeding with TCR-based therapies.

In conclusion, we identified tumor-reactive T cells residing in the bone marrow of pediatric patients with fusion-driven acute leukemias at diagnosis and relapse. Furthermore, these T cells were readily expanded and showed reactivity based on immune activation markers and their cytotoxicity against leukemic blasts, in nearly all samples. We identified TCRs selective for neoepitopes present on the *KMT2A::AFF1* fusion, but these clones were only identified at timepoints while off therapy (e.g., diagnosis or relapse). Altogether, our findings suggest that adoptive transfer of expanded tumor-reactive T cells and/or T cells containing neoantigen-specific TCRs holds promise as a novel therapeutic for fusion-derived pediatric leukemias and that developing a vaccine targeting these neoantigens is likely to improve outcomes.

### Study approval

All methods were performed in accordance with relevant guidelines and regulations. The use of leukemia specimens was approved by the Institutional Review Board of St. Jude Children’s Research Hospital, Pro00006791. Informed consent was obtained from the patients, parents, or guardians, as appropriate. The murine studies were approved by the St Jude Children’s Research Hospital Animal Care and Use Committee and carried out according to Office of Laboratory Animal Welfare guidelines.

## Supplementary information

Supplementary Figures

Supplementary methods

Supplementary tables

## Data Availability

All data produced in this study are presented within the published article and its accompanying Supplementary Information files.
