# Supplementary material for: Endogenous T cell responses to fusion-derived neoantigens in pediatric acute leukemias
Source: Leukemia. 2025 Jul 24;39(10):2419–31. doi: 10.1038/s41375-025-02710-7 (PMC12463655; doi:10.1038/s41375-025-02710-7)
Supplement: Supplementary file 1 — Supplementary Figures [file 41375_2025_2710_MOESM1_ESM.pdf]

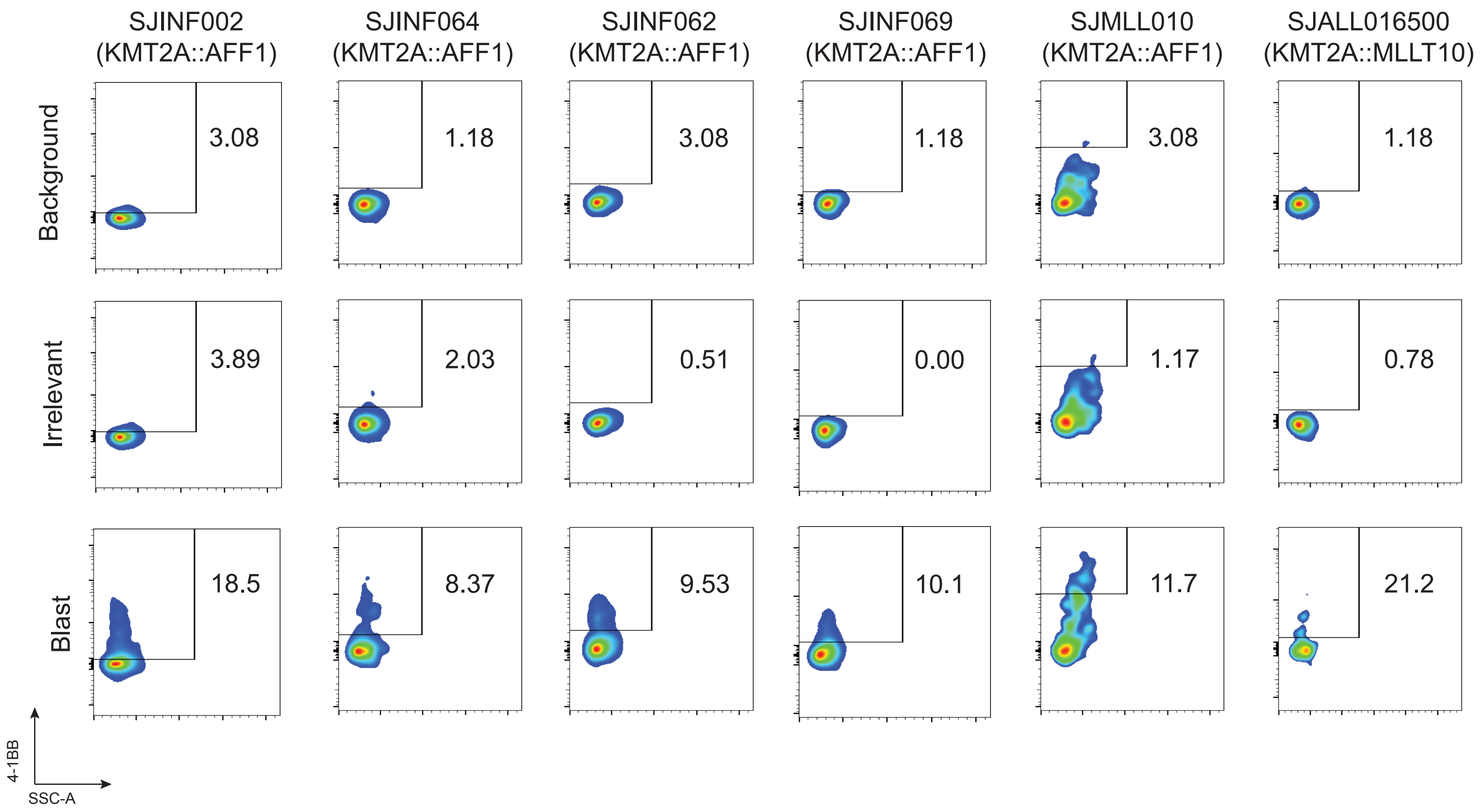

Supplemental Figure 1. T cell response on ALL. Representative co-culture expanded T cells and leukemia blasts -/+ IFN-γ pre-treatment. The reactivity is indicated by flow cytometry detection on 4-1BB upregulation on CD8+ T cells. Irrelevant sample is K562.

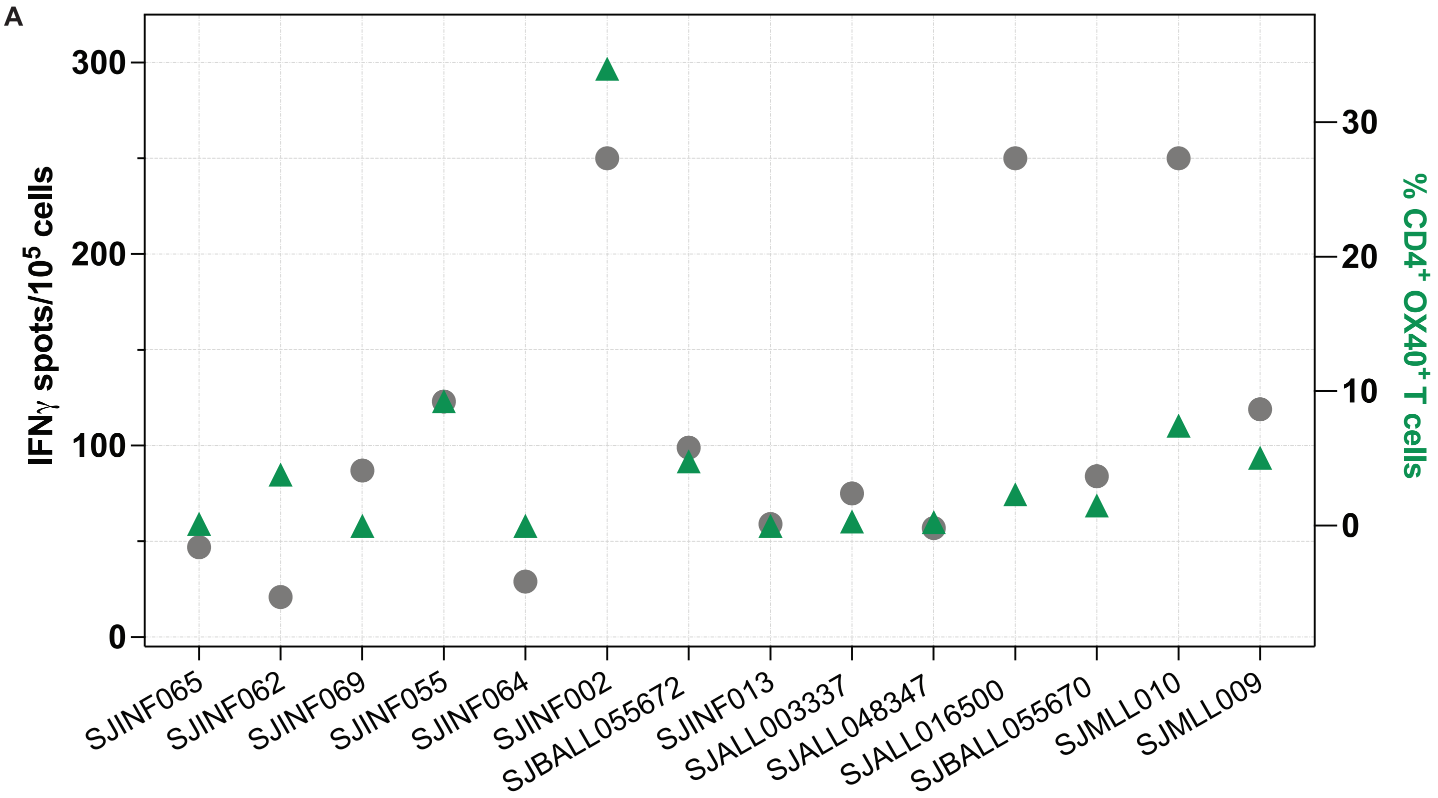

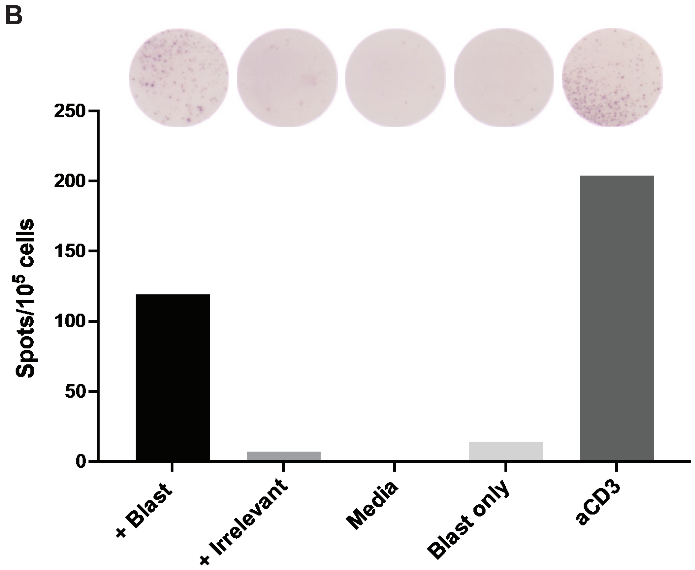

Supplemental Figure 2. Autologous ALL recognition by the expanded T cells. (A) Reactivity of expanded T cells on autologous ALL demonstrated by IFN-γ ELISPOT and flow cytometry on OX40+ CD4+ T cells. The values are normalized to their corresponding background control. (B) SJMLL009 showed the reactivity by IFNγ release despite negativity on CD4 or CD8 activation markers.

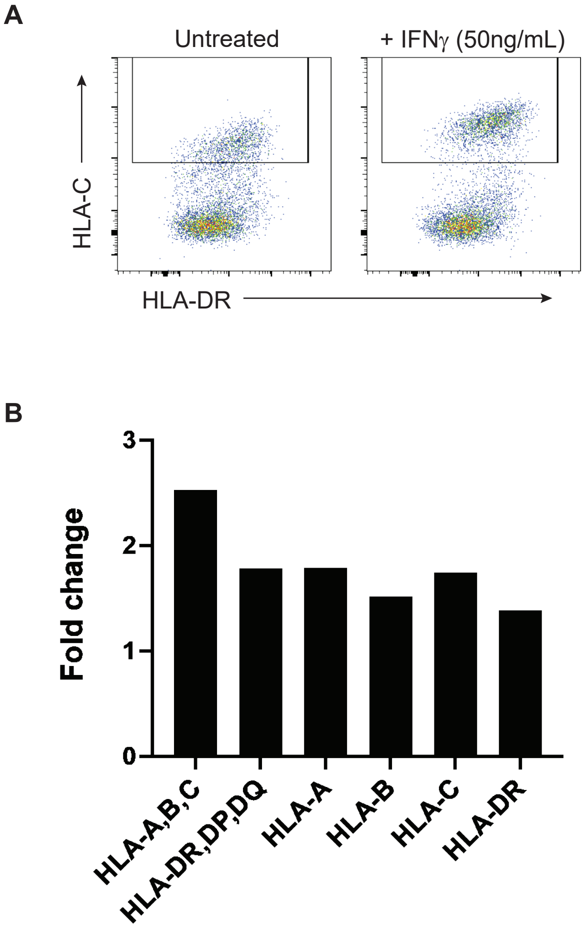

Supplemental Figure 3. MHC expressions on SJALL048347 leukemia blasts upon IFN-γ treatment. (A) Flow cytometry HLA-C and HLA-DR expressions on gated CD19+ SJALL048347. (B) Fold change MHC expression changes normalized to untreated blasts. The values are based on MFI.

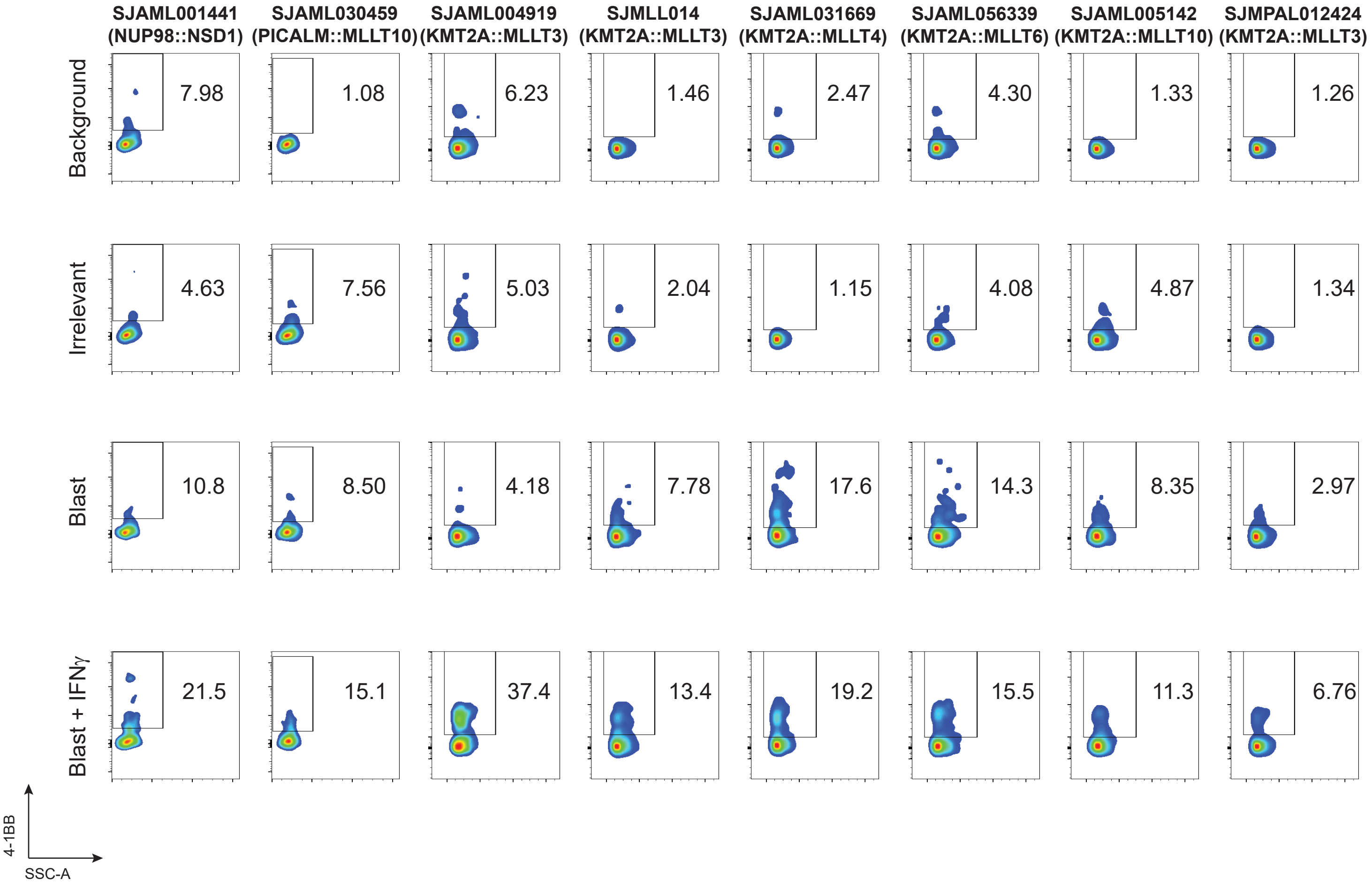

Supplemental Figure 4. T cell response in AML and MPAL requires IFN-γ pre-treatment. Representative co-culture expanded T cells and leukemia blasts -/+ IFN-γ pre-treatment. The reactivity is indicated by flow cytometry detection on 4-1BB upregulation on CD8+ T cells. Irrelevant sample is K562 cells.

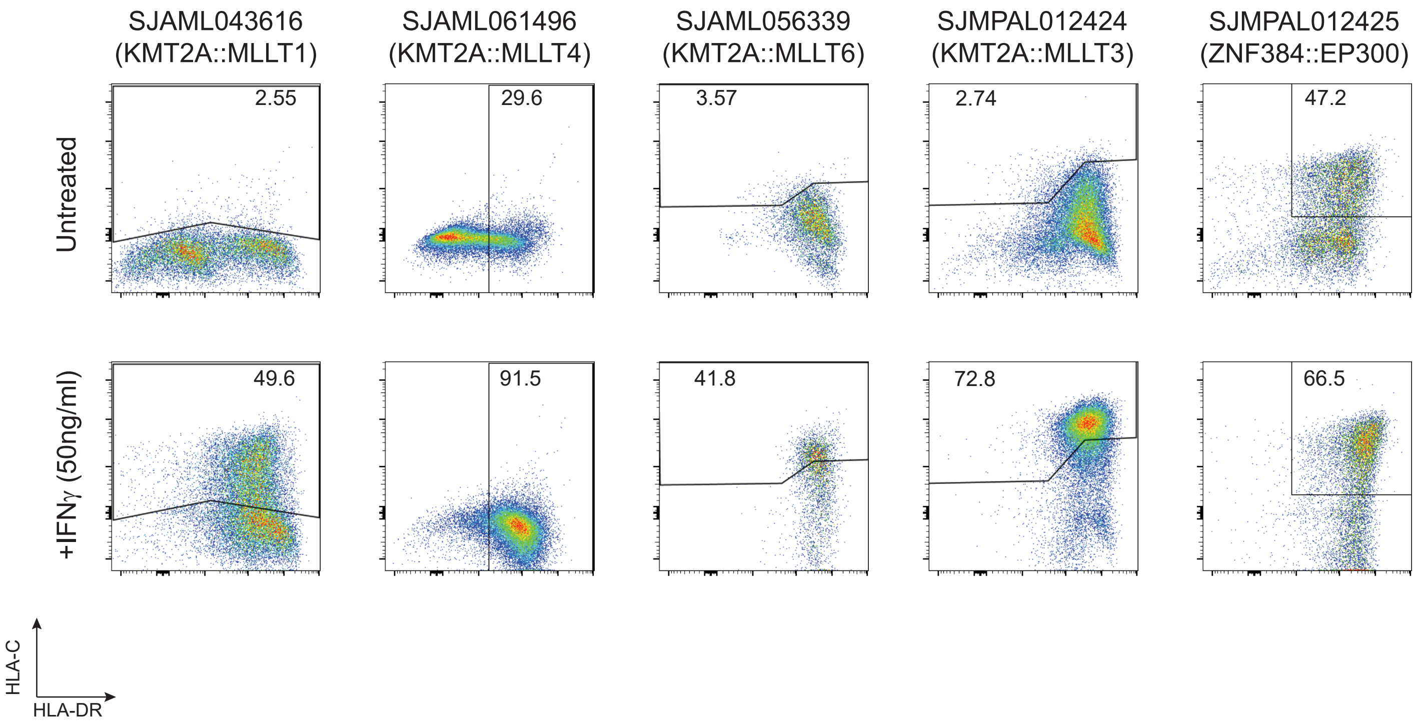

Supplemental Figure 5. Representative flow cytometry MHC expressions primary AML and MPAL cells upon IFN-γ treatment. Flow cytometry analysis HLA-C and HLA-DR expressions on representative AML and MPAL samples. Upregulation HLA alleles are observed on either marker. Population gating is drawn based on the distinct population after the treatment.

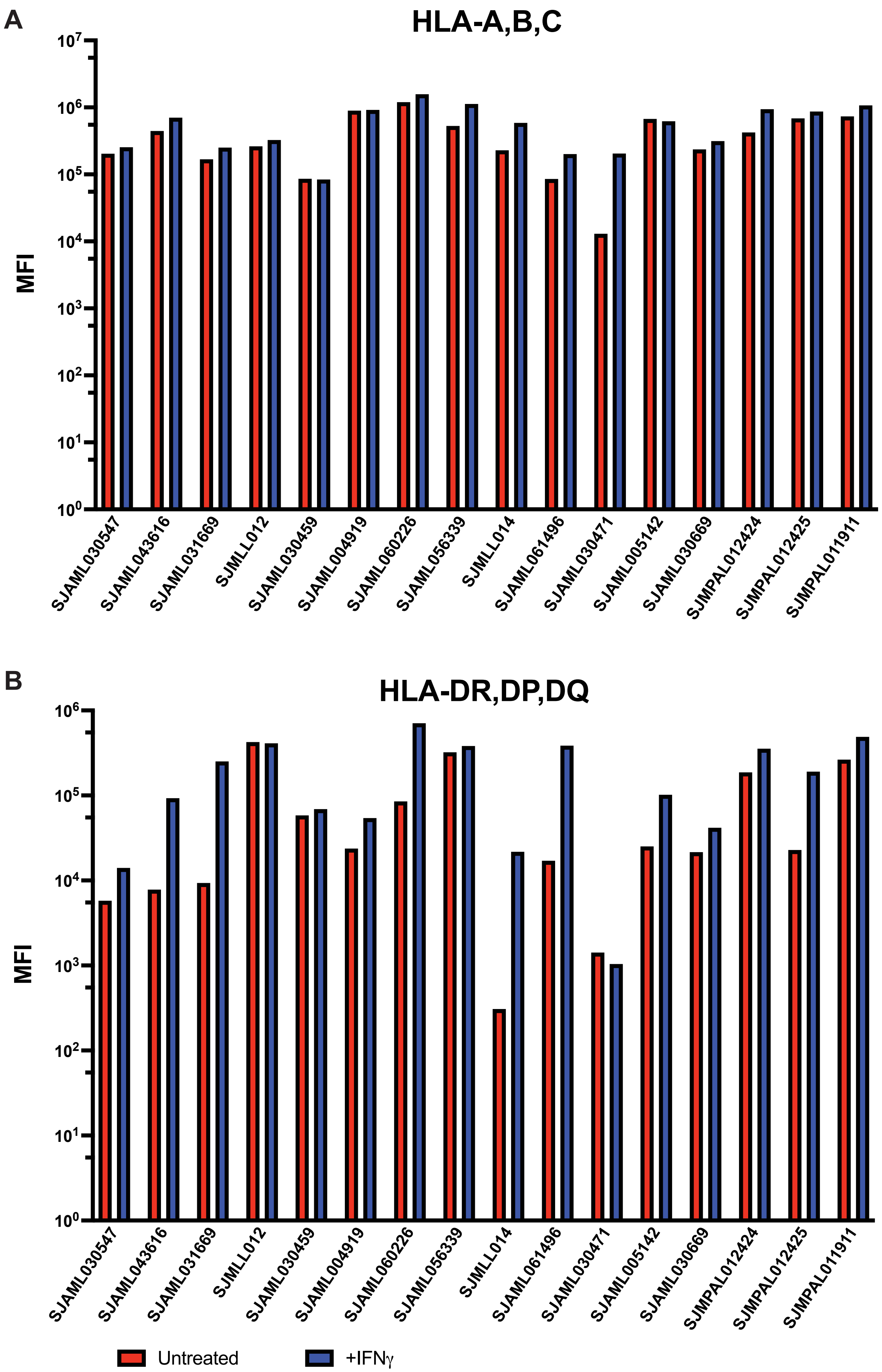

Supplemental Figure 6. MHC expressions on primary AML and MPAL cells upon IFN-γ treatment. Mean fluorescent intensity (MFI) expressions of MHC class I (A) and class II (B) on AML and MPAL samples after 50 ng/ml IFN-γ pre-treatment for 72 h.

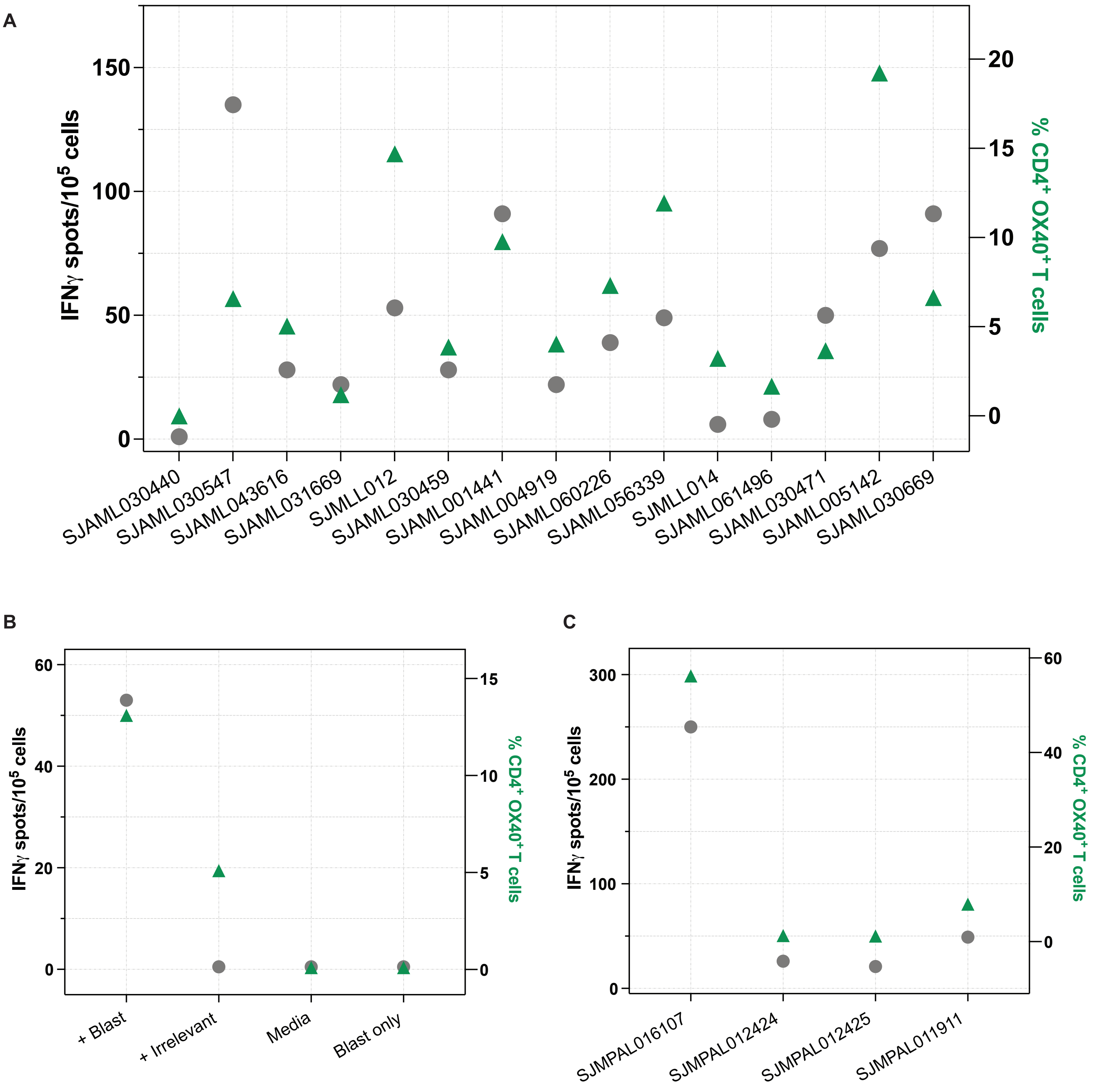

Supplemental Figure 7. Autologous AML and MPAL recognition by the expanded T cells. (A) Reactivity of expanded T cells on autologous AML demonstrated by IFN-γ ELISPOT and flow cytometry on OX40+ CD4+ T cells. (B) Sample SJMLL012 showed reactivity indicated by IFN-γ secretion and OX40 on CD4+ cells, despite no changes in 4-1BB expression. (C) Reactivity on autologous MPAL, as demonstrated by IFN-γ ELISPOT and flow cytometry on OX40+ CD4+ T cells. The values are normalized to their corresponding background control.

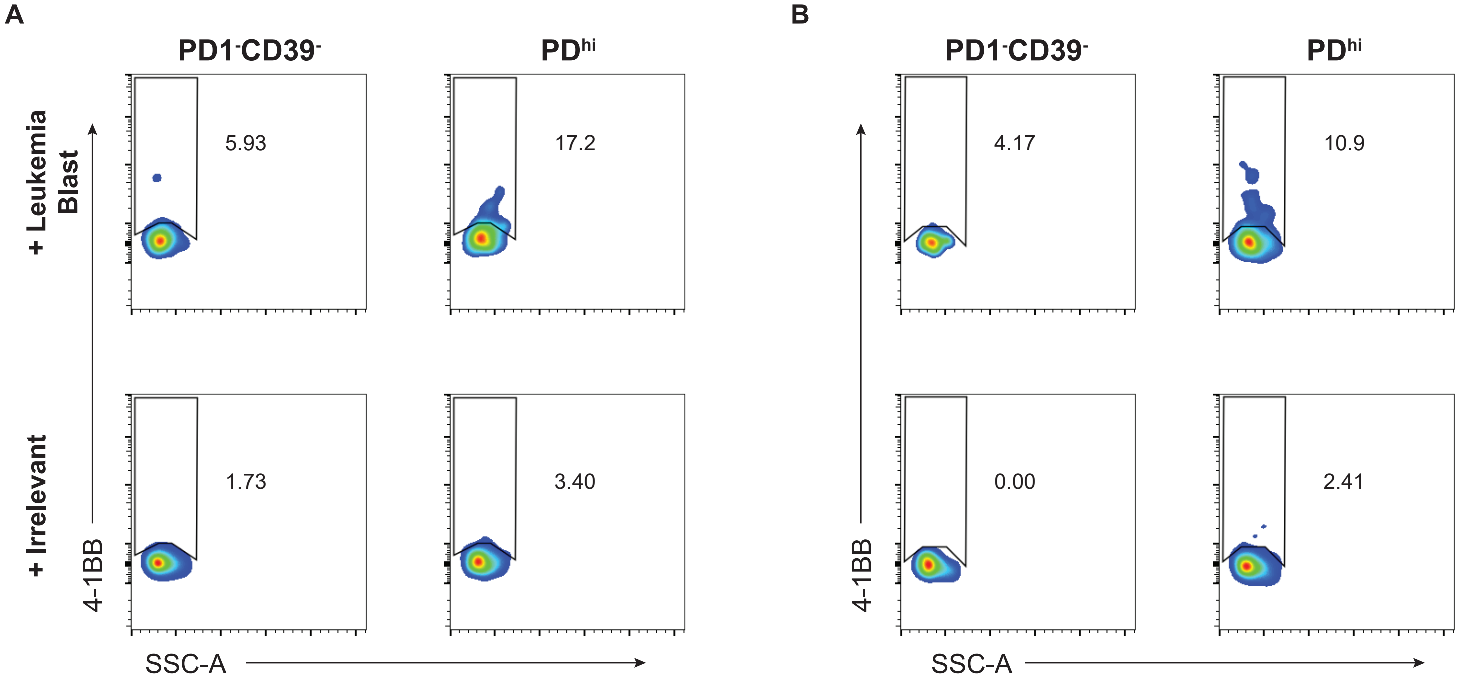

Supplemental Figure 8. Reactivity of CD39 and PD1 T cell subsets from SJAML001441 to leukemia blasts. Co-culture sorted T cell subset populations and autologous blasts from SJAML001441. Sorted populations were expanded for 14 days and co-culture with the blasts for 20 h. T cell recognition was measured by 4-1BB expression on CD8 (A) and CD4 (B). Population PD1$^-$ CD39$^+$ SJAML001441 was not included due to low number of cells.

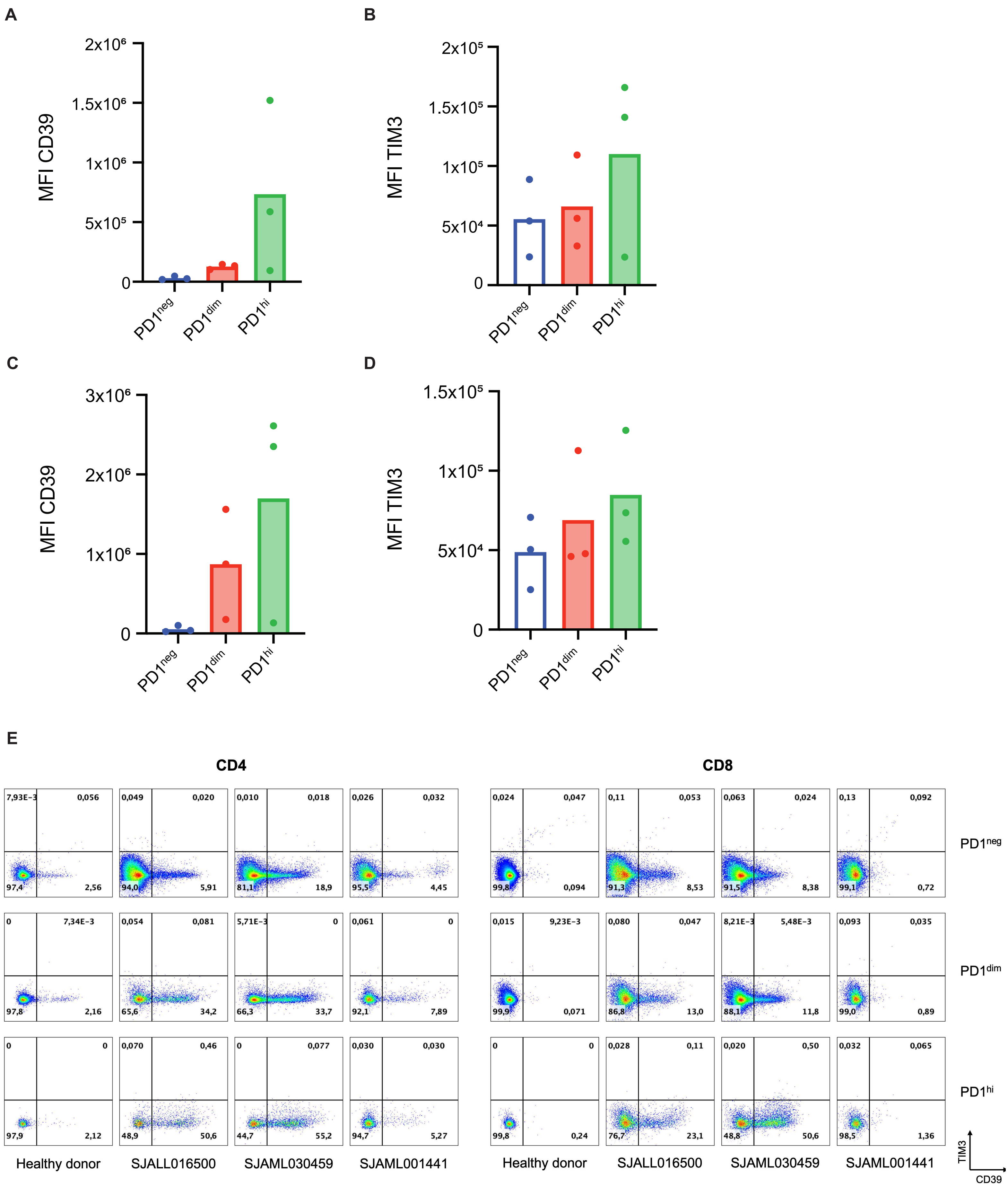

Supplemental Figure 9. Co-expression markers on PD1 subsets. The plots display mean fluorescent intensity (MFI) of CD39 and TIM3 expressions on CD8 (A, B) and CD4 (C, D), grouped by PD1^{neg}, PD1^{dim}, and PD1^{hi} populations from patients SJALL016500, SJAML030459, and SJAML001441. (E) Dot plots showing co-expression of CD39 and TIM3 on CD4 (left) and CD8 (right) T cells, stratified by PD1 expression level (PD1^{neg}, PD1^{dim}, PD1^{hi}).

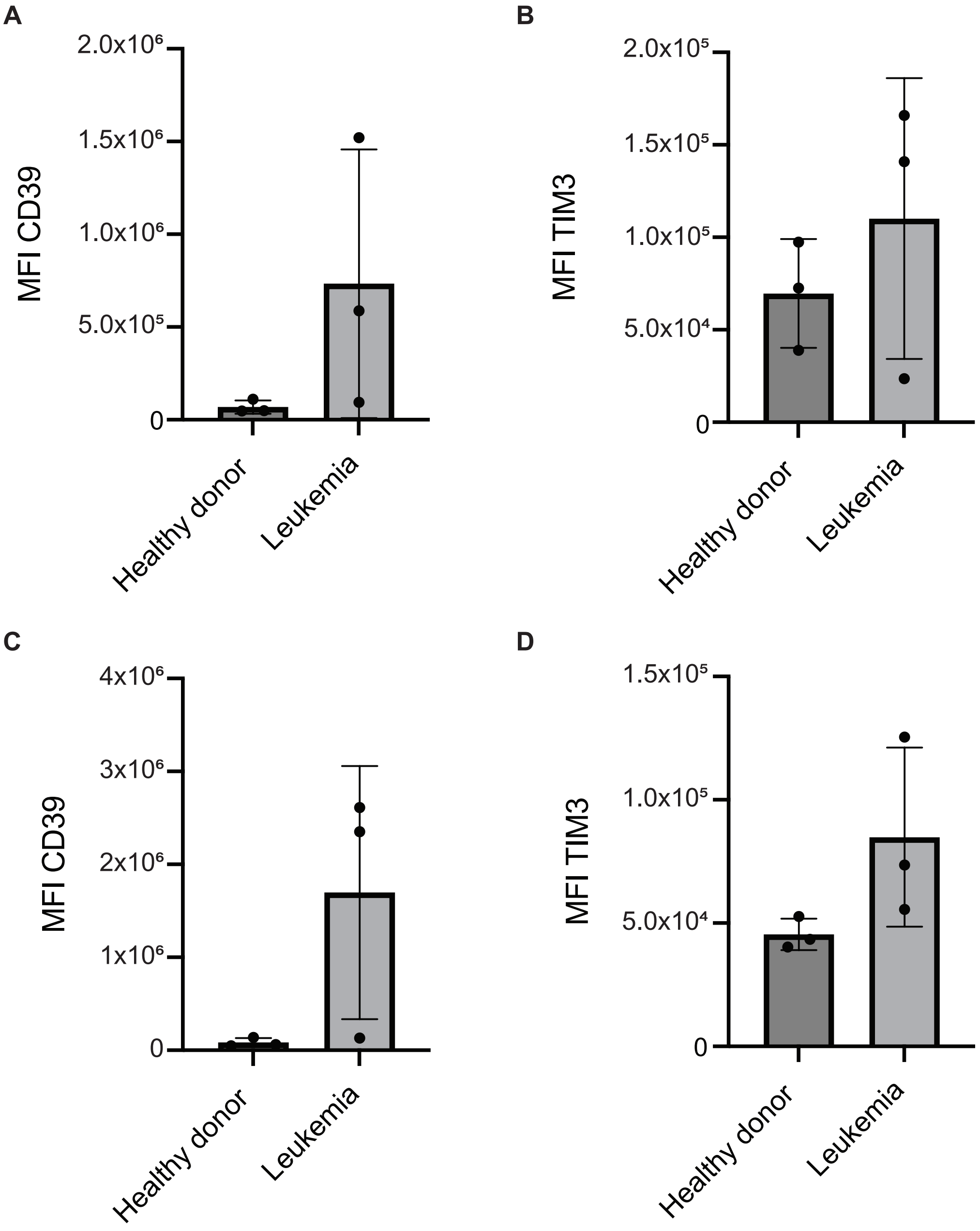

Supplemental Figure 10. CD39 and TIM3 expressions on PD1hi subset from leukemia patients and healthy donors. The plots display mean fluorescent intensity (MFI) of CD39 and TIM3 expressions on PD1^{hi} subset of CD8 (A, B) and CD4 (C, D). Leukemia samples are SJALL016500, SJAML030459, and SJAML001441.

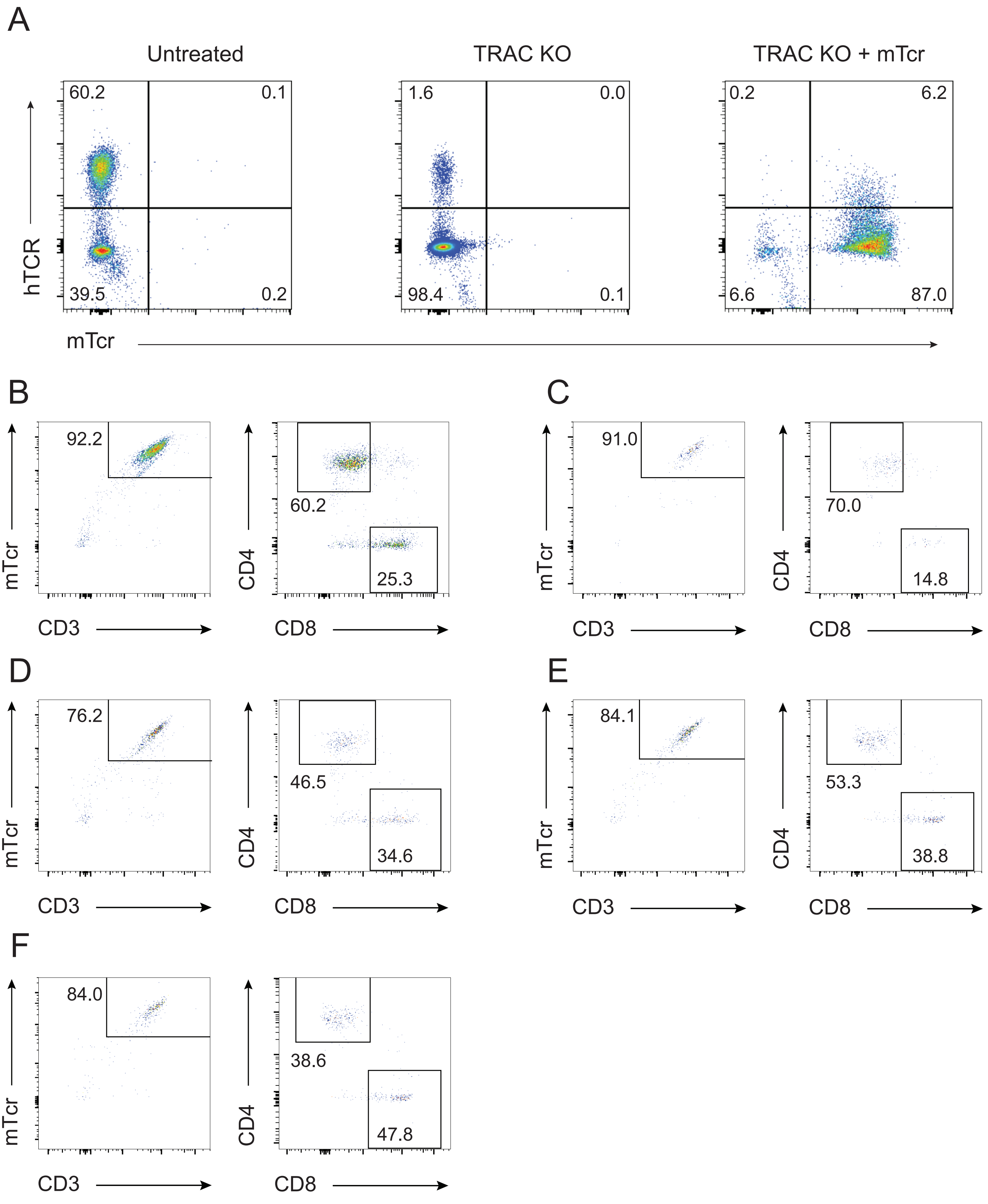

Supplemental Figure 11. Candidate TCR over-expression on endogenous TCR knockout healthy donors. (A) Untreated allogeneic PBMC is indicated on the left panel. The material was treated with T cells activation by anti-CD3 and anti-CD8 followed by CRISPR TRAC knockout; middle panel. Subsequently, the cells were retrovirally transduced with the candidate TCR which using mouse constant region, detected by mouse Tcr antibody; right panel. TCR over-expressions on the reactive TCRs are shown, including SJINF002_2 (B), SJINF013_2 (C), SJAML001441_2 (D), SJAML030459_3 (E), and SJAML030459_5 (F)

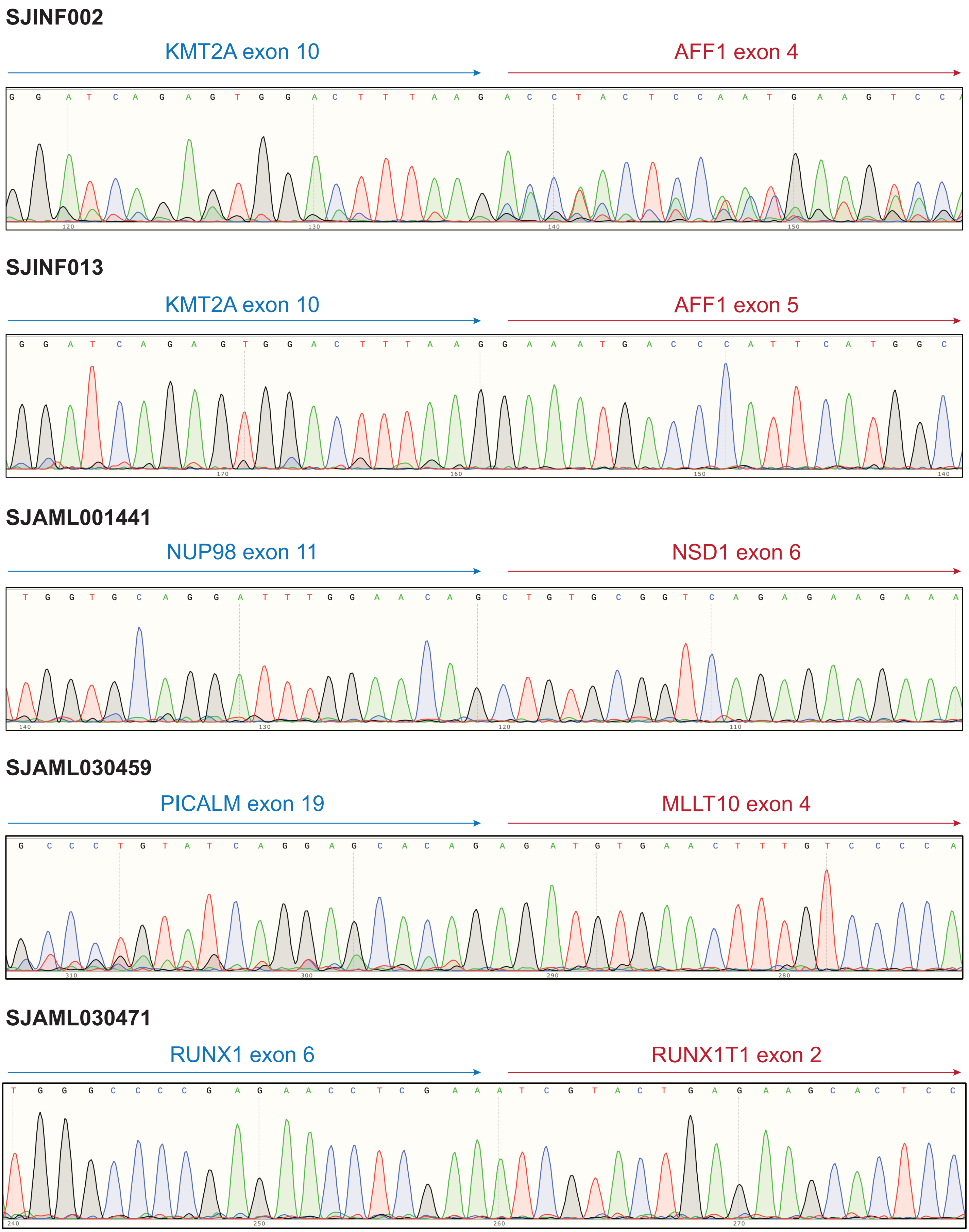

Supplemental Figure 12. Fusion gene breakpoint sequences. Sanger sequencing fusion gene breakpoints on cDNA leukemia blasts.

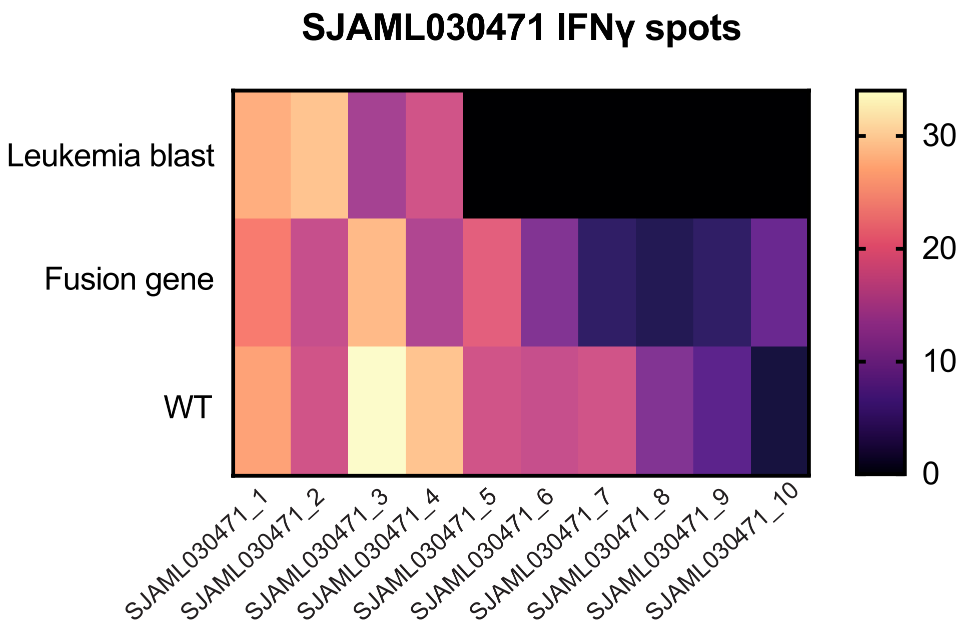

Supplemental Figure 13. Screening candidate TCR SJAML030471's reactivity to primary leukemia blasts and their specific fusion gene presented by patient antigen-presenting cells. TCRs were over-expressed on TRAC knockout allogeneic healthy donor cells. They were co-cultured with primary leukemia materials to evaluate the reactivity to the blasts. Secondly, antigen-presenting cells from the patients were electroporated with their corresponding fusion gene in RNA minigene format. TCR's specificity to the fusion genes were assessed by coculturing with the electroporated cells.

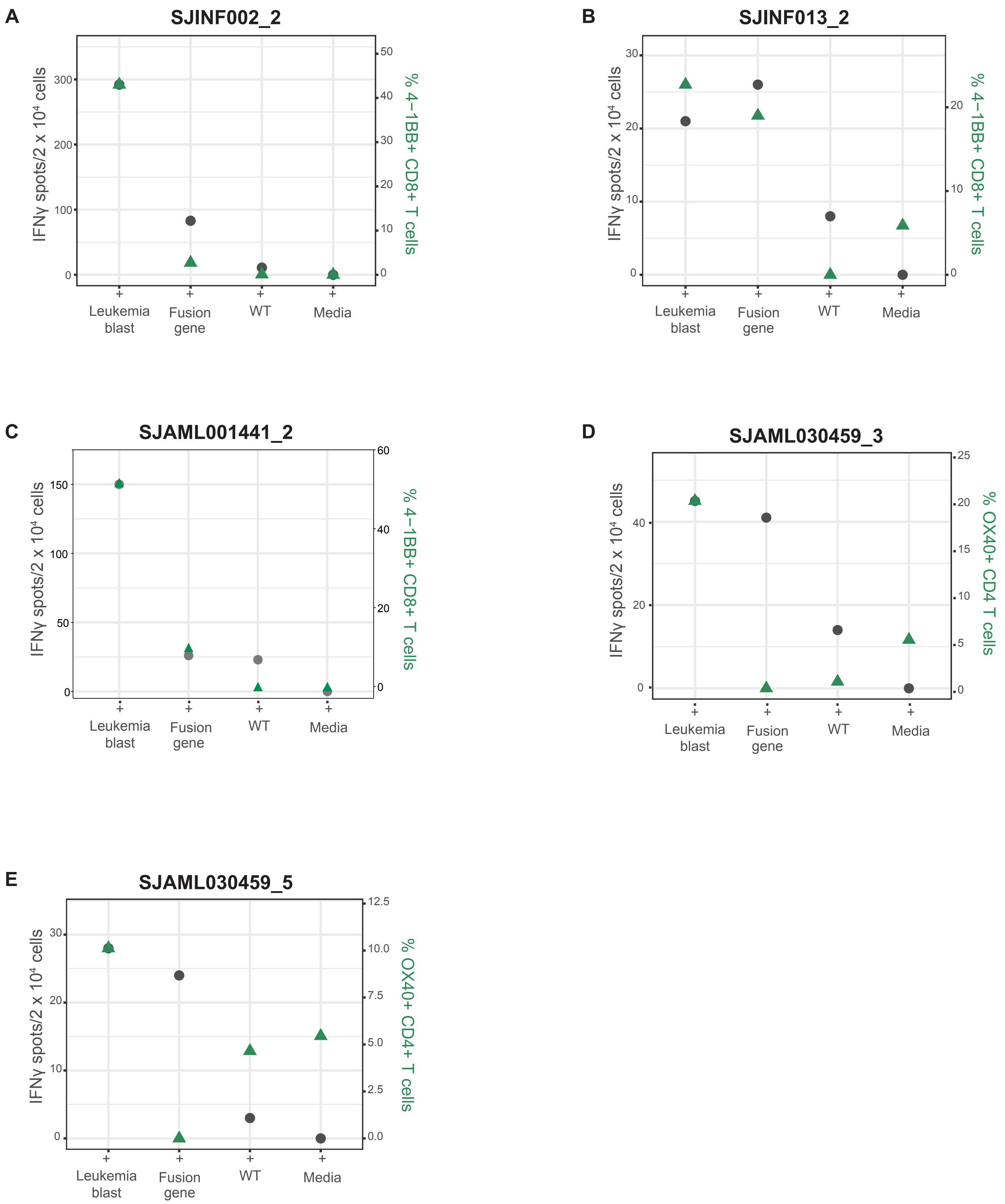

Supplemental Figure 14. TCR reactivity to leukemia blasts and fusion gene neoantigen. Co-culture of TCRs that were reactive to leukemia blasts and fusion genes presented by patient-derived APCs. B cells were used as APCs for sample SJINF013, and $CD4^+$ T cells for other samples. The reactive TCRs included TCR SJINF002 (_2 indicates ranked 2 by frequency) (A), TCR SJINF013_2 (B), TCR SJAML001441_2 (C), TCR SJAML030459_3 (D), and SJAML030459_5 (E). The reactivity was measured by IFN-γ ELISPOT (left axis) and flow cytometry upregulation of OX40 or 4-1BB (right axis). The fusion genes were *KMT2A* exon 10–*AFF1* exon 4 (SJINF002), *KMT2A* exon 10–*AFF1* exon 5 (SJINF013), *NUP98* exon 11–*NSD1* exon 6 (SJAML001441), *PICALM* exon 19–*MLLT10* exon 4 (SJAML030459). Wild type (WT) refers to the germline *KMT2A*, *NUP98*, and *PICALM* sequences, respectively.

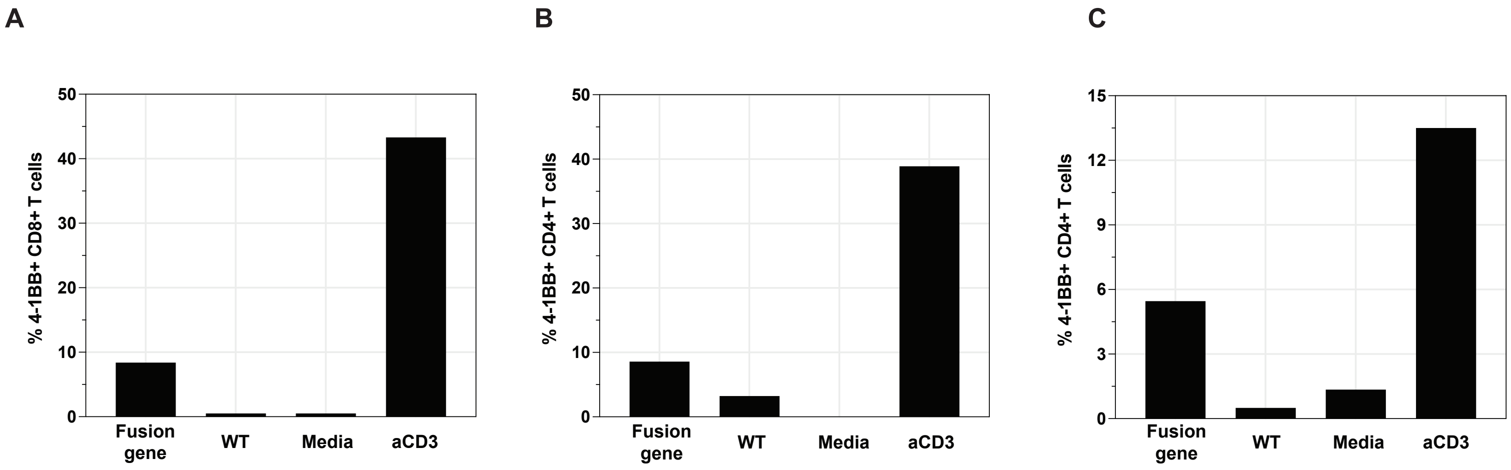

Supplemental Figure 15. Reactivity of candidate TCRs to fusion gene. TCRs were over-expressed on TRAC-knockout allogeneic healthy donor cells. Antigen-presenting cells derived from the patients were electroporated with the corresponding fusion gene in RNA minigene format. The reactivity of the co-cultured components was observed as follows: (A) TCR SJINF002_2 to *KMT2A::AFF1*, (B) TCR SJINF013_2 to *KMT2A::AFF1*, and (C) TCR SJAML001441_2 to *NUP98::NSD1*.

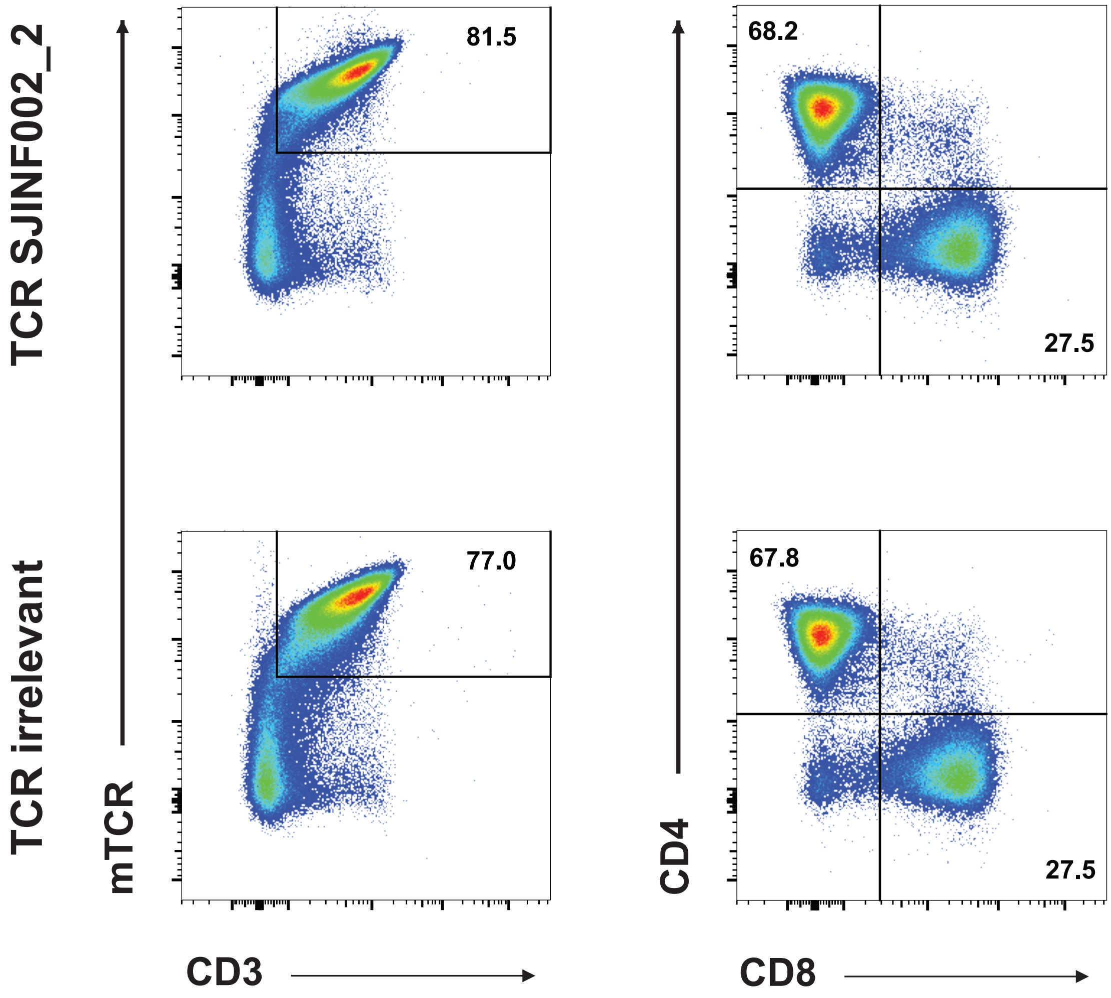

Supplemental Figure 16. TCR over-expression in TRAC knockout healthy donor cells for *in vivo* study. CRISPR-mediated TRAC knockout was performed on healthy donor PBMCs, followed by retroviral transduction with either SJINF002_2 or irrelevant TCR. The expression was detected in both CD4 and CD8 T cells.

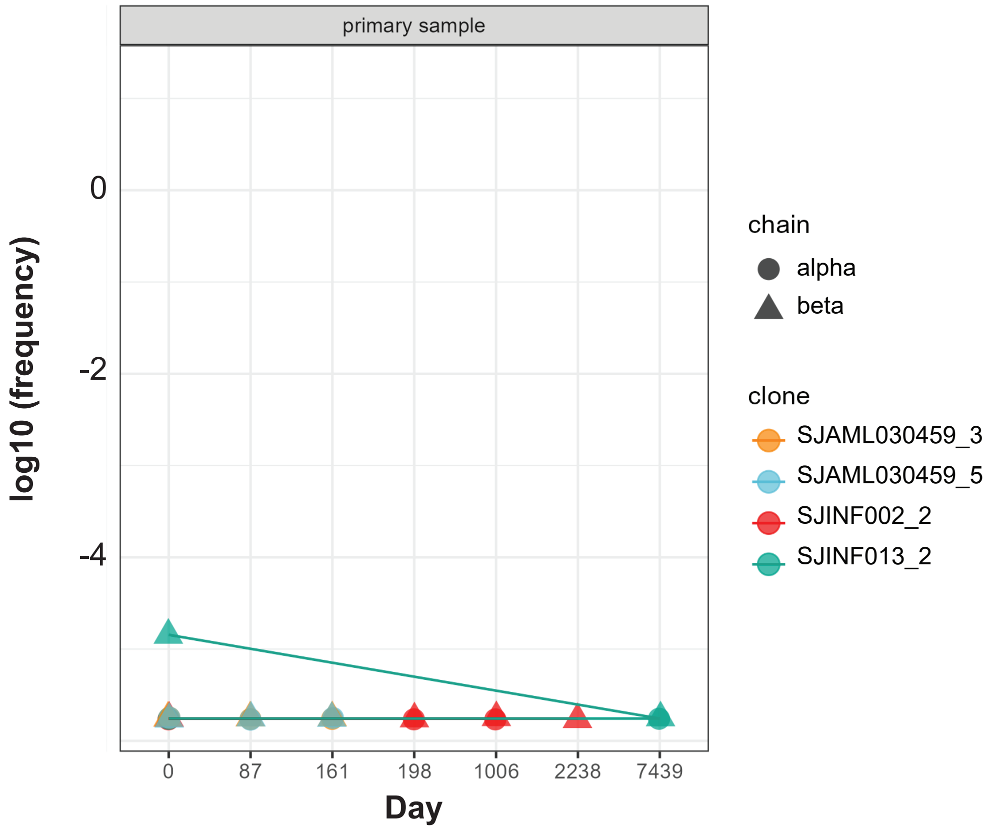

Supplemental Figure 17. Frequency reactive TCRs at different time points. The frequencies of reactive TCR clonotypes were evaluated across various patient biopsy collection time points. For the SJAML030459 sample, biopsies were collected at Days 0, 87, and 161. The SJINF002 was analyzed at Days 0, 198, 1006, and 2238, while the SJINF013 sample was evaluated at Days 0 and 7439. None of the clonotypes were detected at any time points, except for SJINF013, which showed minimal detection at diagnosis.

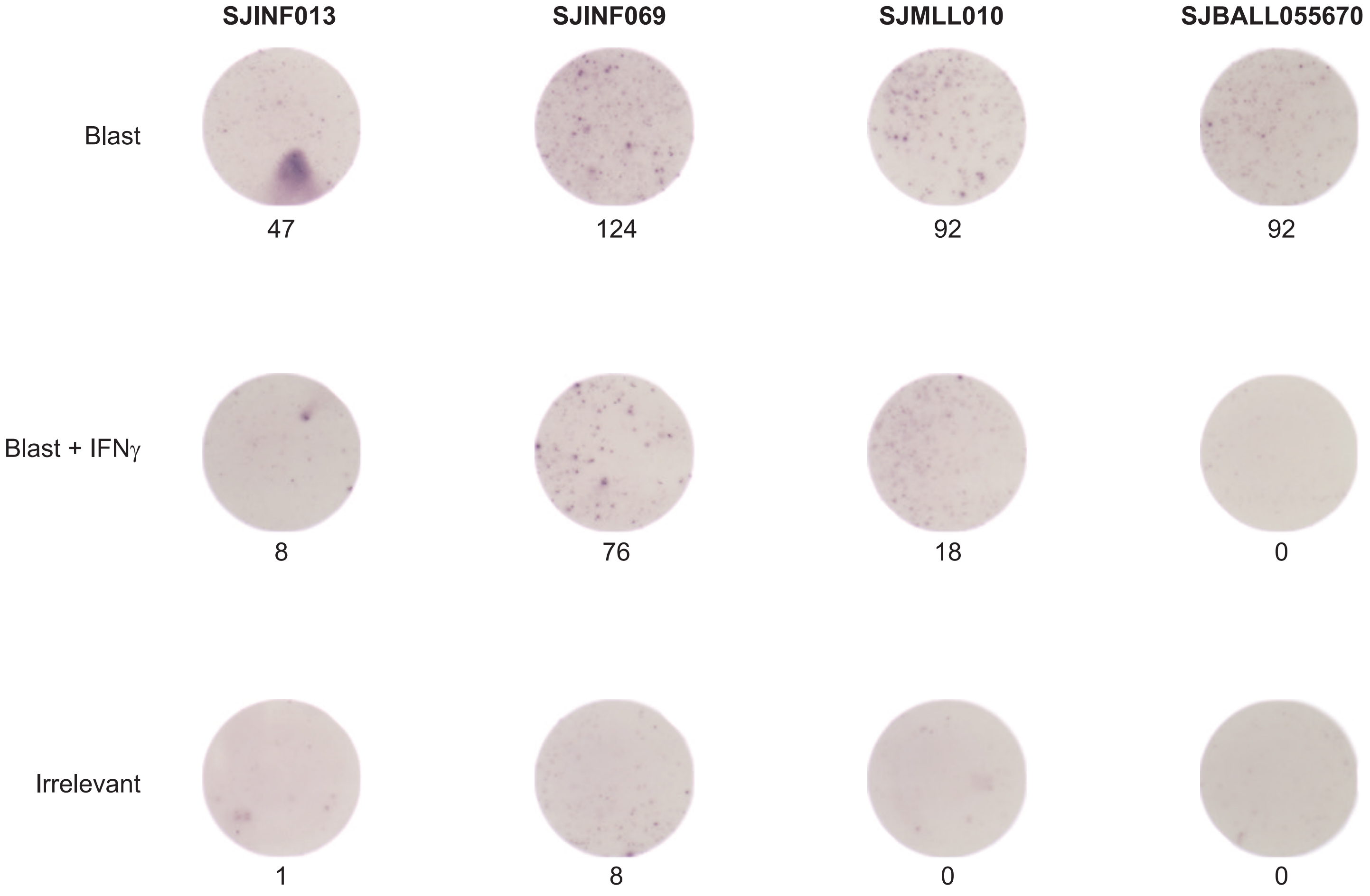

Supplemental Figure 18. T cell reactivity on ALL blasts with IFN-γ pre-treatment. ALL blasts were pre-treated with 50 ng/ml IFN-γ for 72 hours then coculture with autologous expanded T cells. The untreated blasts (top row) showed higher IFN-γ spots compared with the pre-treated blasts. Irrelevant control is K562 cells.
