# Supplementary material for: Endogenous T cell responses to fusion-derived neoantigens in pediatric acute leukemias
Source: Leukemia. 2025 Jul 24;39(10):2419–31. doi: 10.1038/s41375-025-02710-7 (PMC12463655; doi:10.1038/s41375-025-02710-7)
Supplement: Supplementary file 2 — Supplementary methods [file 41375_2025_2710_MOESM2_ESM.docx]

**Methods**

***Leukemia specimens***

The leukemia specimens were used in accordance with the ethical approval granted by the Institutional Review Board of St. Jude Children’s Research Hospital. Informed consent was obtained from the patients, parents, or guardians, as appropriate. Bone marrow or peripheral blood was collected at diagnosis and/or relapse. Mononuclear cells were isolated by density-gradient centrifugation and cryopreserved until analysis. The patient characteristics are provided in Table 1.

***PDX specimens***

Leukemic PDX samples (SJINF002, SJAML001441, and SJAML030459) were obtained through the St. Jude Children’s Research Hospital Public Resource of Patient-derived and Expanded Leukemias (PROPEL) (https://propel.stjude.cloud). Briefly, PDX were established by tail vein injection of primary leukemia cells into eight-twelve-week-old sub-lethally irradiated (250 Rad) NOD.Cg-*Prkdc*^scid^ *Il2rg*^tm1Wjl^ /SzJ (NSG) mice (The Jackson Laboratory) for ALL and NOD.Cg-*Prkdc*^scid^ *Il2rg*^tm1Wjl^ Tg(CMV-IL3,CSF2,KITLG)1Eav/MloySzJ (NSG-SGM3) for AML^1^ (The Jackson Laboratory). Spleen cells harvested from engrafted mice were used for expansion in subsequent passages. The level of engraftment was monitored by monthly retro-orbital bleeds and flow cytometric analysis of human CD45 positive cells. The study was approved by the St Jude Children’s Research Hospital Animal Care and Use Committee and carried out according to Office of Laboratory Animal Welfare guidelines.

***Ex vivo* *culture of ALL, AML, MPAL blasts***

Prior to T-cell reactivity evaluation, leukemia blasts from all patients were initially expanded by co-culture with mesenchymal stem cells, as previously described^2^. In brief, mesenchymal stem cells were seeded at a concentration of 10^4^ cells/cm^2^ in RPMI supplemented with 20% fetal bovine serum (FBS) and 1 µM hydrocortisone 48 hours before adding leukemia cells. The media was aspirated, and leukemia cells were added at a concentration of 1-2 × 10^6^ cells/mL in SFEM II media. Culture media were supplemented with 20 ng/mL interleukin 3 (IL3) and 10 ng/mL IL7 for ALL samples, or with SCF, TPO, IL3, IL6, and FLT3L (10 ng/mL each) for AML and MPAL samples. After 7 days in culture, samples were transferred to a new layer of feeder cells.

***In vitro T-cell expansion***

T cells were expanded based on a rapid expansion protocol (REP) method^3^. In brief, T cells were maintained in culture with irradiated (5,000 rad) allogeneic peripheral blood mononuclear cells (PBMC) from 3 healthy donors. They were maintained in culture media containing 30 ng/mL anti-CD3 OKT3 and 3,000 IU/mL IL2 for 6 days and then this media was replaced IL2 (3,000 IU/mL) containing fresh media every 2 days thereafter. At Day 15, the expanded T cells were either used in co-culture assays or cryopreserved.

***Generation of B cells***

B cells were used as APCs and were generated from PBMCs obtained during the patient’s remission state. The cells were isolated using CD19^+^ microbeads (Miltenyi Biotec) and maintained in culture with irradiated NIH3T3 CD40L feeder cells, as previously described^3,4^. Cells were expanded for 5 days and re-stimulated for 3 rounds in B-cell media; Iscove's Modified Dulbecco's Medium (IMDM; ATCC) containing 10% human serum AB, 100 U/mL penicillin, 100 µg/mL streptomycin, 2 mM L-glutamine, and 200 U/mL IL4 (Peprotech). Following the expansion, the cells were either used in co-culture assay or cryopreserved. When cryopreserved samples were used, the B cells were thawed and rested in B-cell media for 16–24 hours before the co-culture assay^3^.

***Flow cytometry and cell sorting***

Cells were subjected to FACS analysis by incubation with a mix of fluorescently labeled antibodies for 30 minutes at 4°C after blocking Fc receptors with Human TruStain FcX solution (BioLegend) for 10 minutes at 4°C. Flow cytometry analysis was performed using a Cytek Aurora spectral cytometer. Cells were sorted using a Bigfoot spectral cell sorter (ThermoFisher). Data were analyzed using FlowJo software. Positivity was considered when the readout values were greater than a 2-fold increase over the irrelevant or wild-type control and more than 0.5%.

Antigen-reactive T lymphocytes were sorted based on CD8^+^ 4-1BB^+^ and CD4^+^ 4-1BB^+^ or CD4^+^ OX40^+^, as previously described^5,6^. Single cells were sorted into a 384-well plate and subsequently processed for targeted TRA and TRB sequencing. CD39^+^ and PD1^+^ T cells were sorted from primary leukemia blasts. PD1^+^ T cells were defined as the top 20% of the CD3^+^ population. The enriched cells were expanded using the REP culture system.

Antibodies used for T-cell reactivity assessment, cell sorting, and PD1^+^ population characterization included CD3 APC/H7 (RRID: AB_1645475), CD4 Alexa Fluor 700 (RRID: AB_2563150), CD4 PE (RRID: AB_395752), CD8 PE/Cy7 (RRID: AB_396852), OX40 FITC (RRID: AB_396160), 4-1BB APC (RRID: AB_398477), CD39 BV421 (RRID: AB_2564575), PD1 PE (RRID: AB_940483), TIM3 BV650 (RRID: AB_2565829), and mTCRB PE (AB_466066). Antibodies used for MHC expression analysis included CD19 PE/Cy5 (RRID: AB_314240), CD33 Alexa Fluor 647 (RRID: AB_2927892), CD33 PE (RRID: AB_2566106), HLA-A,B,C PE/Cy5 (RRID: AB_314877), HLA-DR,DP,DQ APC/Fire 750 (RRID: AB_2750314), HLA-A BV711 (RRID: AB_2917819), HLA-B PE (RRID: AB_2916506), HLA-C Alexa Fluor 647 (RRID: AB_2894582), and HLA-DR (RRID: AB_2561913).

***CRISPR-Cas9 TRAC knockout***

T cells from healthy donor apheresis were activated using anti-CD3/CD28 Dynabeads (ThermoFisher) in RPMI supplemented with 10% FBS, 100 U/mL penicillin, 100 µg/mL streptomycin, 2 mM L-glutamine, 10 mM HEPES, 50 IU/mL IL2, 10 ng/mL IL7, and 10 ng/mL IL15. The cells were seeded at a concentration of 2 × 10^6^ cells/mL and activated for 48 hours. Following incubation, the beads were removed, washed, and resuspended in Buffer P3 Primary Cell Nucleofector (Lonza) at a concentration of 5-10 × 10^4^ cells/mL in 100-µL volume. CRISPR-Cas9 ribonucleoproteins, formulated by complexing 6 µM Cas9 and 6 µM sgTRAC (1:1 ratio), were added to the cells and then electroporated (Lonza, X-unit, EH-115). The cells were recovered by adding RPMI supplemented with 10% FBS and 180 IU/mL IL2 at a concentration of 2 × 10^6^ cells/mL for 16–24 hours before TCR retrovirus transduction.

***TCR retrovirus production and transduction***

TRB and TRA sequences were joined by a furin SGSG P2A linker. Mouse constant regions were used^7–9^. The sequences were synthesized (Twist Bioscience) and cloned into a pSFG retroviral backbone. The co-transfection consisted of 9 µg expression plasmid, 9 µg Peq-Pam, 4 µg RD114, and 60 µL Lipofectamine 2000. The mixture was added to 293T cells in a 10-cm dish format or down-scaled in a 35-mm dish with the same ratio. Supernatant was collected at 24 and 48 hours, filtered using 0.45-µm pore size, and used for transduction or stored at –80°C. Transduced cells were used at Day 7.

***Construction of minigene, in vitro transcription, and electroporation***

Fusion minigenes were constructed by including 100 amino acids flanking the breakpoint and cloned into the pcDNA3.1 vector. The sequences were verified by Sanger sequencing. The vector was linearized and then purified using NucleoSpin Gel and PCR Clean-up (Macherey-Nagel). HiScribe T7 ARCA mRNA Kit, with tailing (NEB) was used to generate *in vitro* transcription RNA per the manufacturer’s instructions. RNA was purified by LiCl_2_ precipitation and resuspended in H_2_O at a concentration of 1 µg/µL.

B cells were washed and resuspended at a concentration of 5-10 × 10^6^ cells in 100 µL optiMEM. Then 8 µg RNA was added to the cells, which were then transferred to a 2-mm gap cuvette and electroporated at 150 V, 20 ms, 1 pulse^10^ using a BioRad Gene Pulser. The B cells were incubated at 37°C, under 5% CO_2_, for 16 hours and used for co-culture assays.

***Generating lentiviral-transduced K562 MHC class II APCs***

The MHC class II machinery cDNA sequences were synthesized by Genscript. The components were generated as 2 separate transgenes, including CD64, CD80, CD83 CD74, and HLA-DM. They were cloned into a pLVX lentiviral backbone and transduced into K562 cells. The cells were stained with fluorescent antibodies, and the population that expressed all components was isolated.

Gene fragments encoding patients’ HLA were acquired from Twist Bioscience. Each HLA sequence was cloned into the pLVX-EF1a-IRES-Puro lentivirus expression vector (Clonetech). Lentivirus was produced by co-transfection of pLVX lentivirus vector containing an HLA insert, psPAX2 packaging plasmid, and pMD2.G envelope plasmid into the 293T packaging cell line by using polyethylenimine (PEI). The cells were washed 4 hours after co-transfection. After 24 and 48 hours, the viral supernatant was collected and filtered. K562 cells expressing MHC class II machineries were subsequently transduced and subjected to puromycin selection for 1 week in IMDM supplemented with 10% FBS. The expression of single HLA molecules was verified by flow cytometry.

***Assessment of T-cell reactivity: IFN-γ ELISPOT assay and detection of activation markers by flow cytometry***

T-cell reactivity was assessed by IFN-γ secretion and upregulation of 4-1BB and OX40 on CD8^+^ and CD4^+^ T cells, respectively^3–5^. 4-1BB acts as a costimulatory molecule that enhances proliferation and survival of activated T cells. Its expression is highly restricted to recent TCR engagement, making it useful marker for identifying and selecting antigen-specific T cells^11^. IFN-γ secretion was measured by ELISPOT. Before the ELISPOT assay was processed, the cells were collected and stained for 4-1BB and OX40 for flow cytometry analysis, as previously described^3,4^. The assessments were performed by co-culture of 100,000 target cells and T cells for 20 hours. The number of T cells differed based on their sources: 100,000 cells were used when expanded T cells from primary leukemia material were used, and 20,000 cells were used when specific candidate TCR T cells were used. T cells and their respective targets were washed to remove excess cytokines before co-culture. The co-cultures were performed in T-cell media without exogenous cytokines. Plate-bound anti-CD3 OKT3 was used as the positive control. Media without T cells, irrelevant TCRs, and irrelevant peptides were used as negative controls. Positivity was considered when the readout values were greater than a 2-fold increase over that of the background and more than 40 spots were detected in the ELISPOT assay. The experiments were performed in duplicate, unless otherwise specified, due to the limited availability of the patient samples.

***In vitro cytotoxicity assay***

*In vitro* cell-mediated killing assay was performed based on CFSE (carboxyfluorescein succinimidyl ester) and live/dead staining^10^. Target cells (3 × 10^4^ cells) were stained with 0.25 µM CFSE by incubating at 37°C, under 5% CO_2_, for 20 minutes. Cells were washed, then co-cultured with T cells at an effector-to-target ratio of 1:1, 2;1, or 8:1. The co-culture was conducted for 18 hours in a 96-well plate. Following incubation, the cells were stained with Live/Dead Ghost Dye Violet 510 (Tonbo) for 30 minutes at room temperature. The cells were then washed and resuspended in staining buffer containing 5 µL CountBright Absolute Counting Beads (ThermoFisher) per well. The samples were analyzed by flow cytometry, and cytotoxicity was calculated from duplicates. Target cells without T cells were used as the negative control, and 50 µg/mL blasticidin was used as the positive control.

***In vivo ALL patient-derived xenograft – T cells model***

Ten weeks old NSG mice (The Jackson Laboratory) were intravenously transplanted with 10^6^ primary SJINF002 blasts. The mice were further bred for three weeks, then 5 × 10^6^ TCR SJINF002_2 and TCR irrelevant were intravenously infused. Leukemia burden (hHLA-DR DP DQ^+^, hCD45^+^, hCD19^+^) and T cells (hCD45^+^, hCD3^+^, hCD4^+^, hCD8^+^, mTcrb^+^) were evaluated by weekly bleeding and FACS analysis. All procedures were performed according to Institutional Animal Care and Use Committee protocols by approved by SJCRH. TCR irrelevant control is T cells expressing TCR specific to RAS G12V mutation (KLVVVGAVGV).

***Single-cell TCR sequencing***

The 4-1BB^+^ CD8^+^ T cells and 4-1BB^+^/OX40^+^ CD4^+^ T cells were individually sorted into 384-well plates, and TCRα and TCRβ chains were amplified by nested PCR using variable and constant region-specific primers. A modification to this protocol was the inclusion of well barcodes in second-round primers. PCR products from all wells on a plate were pooled and indexed for sequencing on Illumina platforms using a KAPA HyperPrep Kit (Roche). The 150-bp paired-end sequencing was performed on an Illumina NovaSeq6000 by the St. Jude Hartwell Center.

***Bulk TCR library prep and sequencing***

TCRα and TCRβ chains were amplified using a 5ʹ Rapid Amplification of cDNA Ends (RACE) with unique molecular identifiers (UMIs) for error correction, essentially as described^12^. RNA was extracted and reverse-transcribed using SmartScribe RT reagent (Takara), and Q5 polymerase (New England Biolabs) was used during first- and second-round amplifications. Barcoded TCRα and TCRβ amplicons generated by the second-round PCR were pooled by equal volume, prepped, and indexed for sequencing on Illumina platforms using a KAPA HyperPrep Kit (Roche). The 150-bp paired-end sequencing was performed on an Illumina NovaSeq6000 by the St. Jude Hartwell Center.

***Processing bulk and single-cell TCR sequencing***

MIXCR (v4.2.0)^13^ using the analyze amplicon routine was used for demultiplexing, alignment, and clonotype assembly beginning from paired-end FASTQ reads using the analyze generic-tcr-amplicon-separate-samples-umi and generic-tcr-amplicon built-ins for bulk and single-cell processing, respectively. Custom R (v4.1.2) scripts were used to determine paired chains from parsed single-cell TCR data.

1 Holmfeldt L, Mullighan CG. Generation of human acute lymphoblastic leukemia xenografts for use in oncology drug discovery. *Curr Protoc Pharmacol* 2015; **68**: 14.32.1-14.32.19.

2 Pal D, Blair HJ, Elder A, Dormon K, Rennie KJ, Coleman DJL *et al.* Long-term in vitro maintenance of clonal abundance and leukaemia-initiating potential in acute lymphoblastic leukaemia. *Leukemia* 2016; **30**: 1691–1700.

3 Gros A, Parkhurst MR, Tran E, Pasetto A, Robbins PF, Ilyas S *et al.* Prospective identification of neoantigen-specific lymphocytes in the peripheral blood of melanoma patients. *Nat Med* 2016; **22**: 433–438.

4 Tran E, Ahmadzadeh M, Lu Y-C, Gros A, Turcotte S, Robbins PF *et al.* Immunogenicity of somatic mutations in human gastrointestinal cancers. *Science* 2015; **350**: 1387–1390.

5 Parkhurst M, Gros A, Pasetto A, Prickett T, Crystal JS, Robbins P *et al.* Isolation of T-Cell Receptors Specifically Reactive with Mutated Tumor-Associated Antigens from Tumor-Infiltrating Lymphocytes Based on CD137 Expression. *Clinical Cancer Research* 2017; **23**: 2491–2505.

6 Yossef R, Tran E, Deniger DC, Gros A, Pasetto A, Parkhurst MR *et al.* Enhanced detection of neoantigen-reactive T cells targeting unique and shared oncogenes for personalized cancer immunotherapy. *JCI Insight* 2018; **3**: e122467.

7 Haga-Friedman A, Horovitz-Fried M, Cohen CJ. Incorporation of transmembrane hydrophobic mutations in the TCR enhance its surface expression and T cell functional avidity. *J Immunol* 2012; **188**: 5538–5546.

8 Cohen CJ, Zhao Y, Zheng Z, Rosenberg SA, Morgan RA. Enhanced antitumor activity of murine-human hybrid T-cell receptor (TCR) in human lymphocytes is associated with improved pairing and TCR/CD3 stability. *Cancer Res* 2006; **66**: 8878–8886.

9 Cohen CJ, Li YF, El-Gamil M, Robbins PF, Rosenberg SA, Morgan RA. Enhanced antitumor activity of T cells engineered to express T-cell receptors with a second disulfide bond. *Cancer Res* 2007; **67**: 3898–3903.

10 Gros A, Tran E, Parkhurst MR, Ilyas S, Pasetto A, Groh EM *et al.* Recognition of human gastrointestinal cancer neoantigens by circulating PD-1+ lymphocytes. *J Clin Invest* 2019; **129**: 4992–5004.

11 Wolfl M, Kuball J, Ho WY, Nguyen H, Manley TJ, Bleakley M *et al.* Activation-induced expression of CD137 permits detection, isolation, and expansion of the full repertoire of CD8+ T cells responding to antigen without requiring knowledge of epitope specificities. *Blood* 2007; **110**: 201–210.

12 Egorov ES, Merzlyak EM, Shelenkov AA, Britanova OV, Sharonov GV, Staroverov DB *et al.* Quantitative profiling of immune repertoires for minor lymphocyte counts using unique molecular identifiers. *J Immunol* 2015; **194**: 6155–6163.

13 Bolotin DA, Poslavsky S, Mitrophanov I, Shugay M, Mamedov IZ, Putintseva EV *et al.* MiXCR: software for comprehensive adaptive immunity profiling. *Nat Methods* 2015; **12**: 380–381.
