# Supplementary material for: Endogenous T cell responses to fusion-derived neoantigens in pediatric acute leukemias
Source: Leukemia. 2025 Jul 24;39(10):2419–31. doi: 10.1038/s41375-025-02710-7 (PMC12463655; doi:10.1038/s41375-025-02710-7)
Supplement: Supplementary file 3 — Supplementary tables [file 41375_2025_2710_MOESM3_ESM.pdf]

Case	Rank	TRAV	TRAJ	CDR3A	TRBV	TRBJ	CDR3B	Frequency (%)
SJINF002	1	TRAV9-2*01	TRAJ54*01	CALSAQGAQKLVF	TRBV3-1*01	TRBJ1-3*01	CASSQDRFSGNTIYF	12.7
	2	TRAV25*01	TRAJ33*01	CSVRRNSNYQLIW	TRBV29-1*01	TRBJ2-7*01	CSVVRGEGYEQYF	11.7
	3	TRAV13-1*01	TRAJ6*01	CAARSGGSYIPTF	TRBV10-3*01	TRBJ2-3*01	CAISGPGGPPDTQYF	4.9
	4	TRAV16*01	TRAJ10*01	CALSGAGGGNKLTF	TRBV27*01	TRBJ2-7*01	CASSLGPSGSSYEQYF	4.4
	5	TRAV9-2*01	TRAJ10*01	CALNRGGGNKLTF	TRBV20-1*01	TRBJ2-7*01	CSAREGTSVTYEQYF	3.4
	6	TRAV35*01	TRAJ31*01	CAGQWNNARLMF	TRBV27*01	TRBJ2-7*01	CASSPSEVSYEQYF	2.4
	7	TRAV38-1*01	TRAJ41*01	CAFMNSNSGYALNF	TRBV7-8*01	TRBJ2-7*01	CASSWTSGRGYEQYF	2.0
	8	TRAV12-2*01	TRAJ4*01	CAVNGGYNKLIF	TRBV15*01	TRBJ2-7*01	CATSRDLSGTQYEQYF	2.0
SJINF013	1	TRAV12-3*01	TRAJ13*01	CAFPPGGYQKVTF	TRBV28*01	TRBJ2-7*01	CASSLELVRYEQYF	34.4
	2	TRAV26-1*01	TRAJ42*01	CIVRVRNYGGSQGNLIF	TRBV28*01	TRBJ2-7*01	CASSQPSGRTYEQYF	11.0
	3	TRAV38-2/DV8*01	TRAJ54*01	CAYRRRQGAQKLVF	TRBV20-1*01	TRBJ2-3*01	CSARDINRAGGTDTQYF	5.8
	4	TRAV19*01	TRAJ8*01	CALTMNTGFQKLVF	TRBV2*01	TRBJ2-5*01	CAGGGLKETQYF	2.6
	5	TRAV17*01	TRAJ56*01	CATDALPGANSKLTF	TRBV14*01	TRBJ2-3*01	CASSQVTDTQYF	2.6
	6	TRAV22*01	TRAJ45*01	CAVGVGGGADGLTF	TRBV20-1*01	TRBJ2-7*01	CSAIEAYEQYF	2.3
	7	TRAV13-1*01	TRAJ5*01	CARTGTGRRALTF	TRBV18*01	TRBJ1-4*01	CASSPAGGGEKLFF	1.9
	8	TRAV9-2*01	TRAJ44*01	CALTGNTGTASKLTF	TRBV9*01	TRBJ1-2*01	CASSVGTPGGGYTF	1.9
SJAML030459	1	TRAV41*01	TRAJ49*01	CALGDLGRNQFYF	TRBV2*01	TRBJ2-7*01	CASTVESSYEQYF	19.1
	2	TRAV17*01	TRAJ49*01	CATDGGGNQFYF	TRBV25-1*01	TRBJ1-2*01	CASSPQGISYTF	16.1
	3	TRAV25*01	TRAJ21*01	CGFNKFYF	TRBV7-8*01	TRBJ2-6*01	CASSLVYMGPTSGANVLTF	7.4
	4	TRAV12-3*01	TRAJ26*01	CAINYGQNFVF	TRBV27*01	TRBJ2-7*01	CASSLWATDSYEQYF	3.4
	5	TRAV34*01	TRAJ53*01	CGAEANSGGSNYKLTF	TRBV6-6*01	TRBJ1-2*01	CASSYGGQGIYGYTF	2.7
	6	TRAV12-2*01	TRAJ11*01	CAGVLGGYSTLTF	TRBV9*01	TRBJ2-1*01	CASSVAMTSNNEQFF	2.0
	7	TRAV19*01	TRAJ42*01	CALSSYGGSQGNLIF	TRBV7-2*01	TRBJ2-4*01	CASSLSGTVAKNIQYF	2.0
	8	TRAV14/DV4*01	TRAJ27*01	CAMREGTNAGKSTF	TRBV6-2*01	TRBJ2-1*01	CASSYGGEQFF	2.0
SJAML001441	1	TRAV17*01	TRAJ45*01	CATDADSGGGADGLTF	TRBV6-5	TRBJ1-1	CASSYSPRTEAFF	54.5
	2	TRAV14/DV4*02	TRAJ16*01	CAMREVRFSDGQKLLF	TRBV19	TRBJ1-4	CASSVGTGSATNEKLFF	6.7
	3	TRAV6*01	TRAJ36*01	CALDMGGANNLFF	TRBV20-1	TRBJ1-2	CSARDPARTTGYTF	6.3
	4	TRAV27*01	TRAJ32*01	CALAWGGATNKLIF	TRBV29-1*01	TRBJ2-3*01	CSATGGTDTQYF	2.5
	5	TRAV12-3	TRAJ44	CAMIAGTASKLTF	TRBV6-1	TRBJ1-2	CASTTGPPSGYTF	1.9
	6	TRAV22	TRAJ26	CAVRDNYGQNFVF	TRBV19	TRBJ1-1	CASSIINTEAFF	1.5
	7	TRAV19	TRAJ48	CALSGDFGNEKLTF	TRBV11-3	TRBJ2-2	CASSLTSGGATGELFF	1.3
	8	TRAV8-3	TRAJ46	CAVGRQSSGDKLTF	TRBV4-1	TRBJ2-3	CASSQDTVTTQYF	1.1
SJAML030471	1	TRAV13-1*01	TRAJ9*01	CAASIATGGFKTIF	TRBV6-5*01	TRBJ2-1*01	CASSYSSPPSYNEQFF	4.6
	2	TRAV38-1*01	TRAJ29*01	CAFMKTGNTPLVF	TRBV13*01	TRBJ2-5*01	CASSSWDSQETQYF	4.6
	3	TRAV29/DV5*01	TRAJ49*01	CAASELQFYF	TRBV7-2*01	TRBJ1-6*01	CASSNTGGFSISYNSPLHF	2.0
	4	TRAV26-2*01	TRAJ37*01	CIHPSERAGSSNTGKLIF	TRBV6-5*01	TRBJ1-6*01	CASSYDPGTASPLHF	2.0
	5	TRAV38-1*01	TRAJ57*01	CAFMSQGGSEKLVF	TRBV28*01	TRBJ2-7*01	CASSLAGASSYEQYF	2.0
	6	TRAV1-2*01	TRAJ29*01	CAGPKDGTPLVF	TRBV20-1*01	TRBJ1-2*01	CSARETGAVGYTF	2.0
	7	TRAV9-2*01	TRAJ53*01	CALSSGGSNYKLTF	TRBV5-5*01	TRBJ2-2*01	CASSLTKDRGTNTGELFF	2.0
	8	TRAV21*01	TRAJ34*01	CAVDTDKLIF	TRBV11-2*01	TRBJ2-1*01	CASTSQAVSYNEQFF	2.0

Patient	MHC I						MHC II											
SJINF002	A*01:01:01:01	A*02:01:01:01	B*08:01:01:01	B*37:01:01:01	C*06:02:01:01	C*07:01:01:01	DPA1*01:03:01:03	DPA1*02:01:02:02	DPB1*01:01:01:01	DPB1*03:01:01:01	DQA1*02:01:01:01	DQA1*05:01:01:02	DQB1*02:01:01:01	DQB1*03:03:02:01	DRB1*03:01:01:01	DRB1*07:01:01:01	DRB3*01:01:02:01	DRB4*01:03:01:02N
SJINF013	A*02:01	A*03:01	B*13:01	B*07:02	C*01:14	C*05:01			DPB1*01:01:03	DPB1*01:01:03			DQB1*06:02:01:01	DQB1*06:02:01:01	DRB1*01:01:01:01	DRB1*01:01:01:01	DRB4*01:02	DRB4*01:02
SJAML001441	A*30:01:01:01	A*34:02:01:01	B*15:10:01:01	B*42:01:01:01	C*03:04:02:01	C*17:01:01:02	DPA1*02:01:08:03	DPA1*02:02:02:04	DPB1*01:01:01:01	DPB1*01:01:01:01	DQA1*04:01:01:05	DQA1*04:01:02:02	DQB1*03:19:01:01	DQB1*04:02:01:08	DRB1*03:02:01:01	DRB1*08:04:01	DRB3*01:62:01:01	DRB3*01:62:01:01
SJHBJ2QVY	A*02:01:01:01	A*02:01:01:01	B*40:01:02:01	B*51:01:01:01	C*03:04:01:01	C*14:02:01:01	DPA1*01:03:01:02	DPA1*02:01:04	DPB1*04:01:01:01	DPB1*13:01:01:02	DQA1*01:01:01:01	DQA1*01:01:01:01	DQB1*05:01:01:03	DQB1*05:01:01:03	DRB1*01:01:01:01	DRB1*01:01:01:01	DRB3*01:01:02:01	DRB3*01:01:02:01

Target fusion gene	Forward	Reverse
KMT2A::AFF1	GCCTCCACCACCAGAATCAG	CGAGCATGGATGACGTTCCT
PICALM::MLLT10	GTTTGTTGGATTCACTCCTTCT	GGACATTATCGGCACCATTAC
NUP98::NSD1	ATTTGGAAGCAGCACAACCAG	TCCTCTTCACAGCGGGAAC
RUNX1::RUNX1T1	CCTGCCCATCGCTTTCAAGGTG	AATAGTGCATGGTCGCTTGCT
